# Comprehensive Analysis of the PANoptosis-Related Genes in Stroke Based on Single-Cell RNA-Seq and Spatial Transcriptomics

**DOI:** 10.1155/mi/5828665

**Published:** 2025-11-04

**Authors:** Wenya Bai, Huan Jiang, Guilin Zhou, Xuelian Li, Jianlin Shao

**Affiliations:** Department of Anesthesiology, First Affiliated Hospital of Kunming Medical University, Kunming 650032, Yunnan, China

**Keywords:** cerebral ischemia-reperfusion injury, ischemia stroke, PANoptosis, single-cell RNA-seq, spatial transcriptomics

## Abstract

**Background:**

PANoptosis is implicated in immunoinflammatory diseases, but its role in cerebral ischemia-reperfusion injury (CIRI) remains unclear.

**Methods:**

We integrated single-cell RNA sequencing (scRNA-seq), spatial transcriptomics (ST), and bulk RNA-sequencing (bulk RNA-seq) datasets to explore PANoptosis-related molecular signatures in ischemic stroke. scRNA-seq identified cellular subpopulations; ST revealed spatial expression. Multimodal intersection analysis (MIA) and high-dimensional weighted gene coexpression network analysis (hdWGCNA) detected PANoptosis-related differently expressed genes (DEGs). Gene Set Enrichment Analysis (GSEA)-identified biomarkers were validated in GSE35338 and GSE137482. Analyses characterized spatiotemporal microglial dynamics. *TNFRSF1A* expression was validated by Western blot.

**Results:**

Nine stromal cell subtypes were mapped across 14 brain regions. Stroke-associated microglial clusters showed microglia-specific dysregulation of PANoptosis regulators (*MCL1*, *TNFRSF1A*, and *STAT3*), with *TNFRSF1A* upregulated in the ischemic core. Altered intercellular communication via SPP1, MIF, FN1, and TNF pathways were observed. Pseudotime analysis revealed dynamic microglial reprogramming. *TNFRSF1A* showed time-dependent upregulation post-CIRI, validated at the protein level.

**Conclusions:**

*TNFRSF1A* acts as a key PANoptosis-related biomarker and suggests microglial subclusters as therapeutic targets in ischemic stroke.

## 1. Introduction

Stroke is a critical cerebrovascular emergency and ranks as the third leading cause of global mortality and a primary contributor to long-term disability [1]. In China, the burden of stroke is escalating due to an aging population, with approximately 2.7 million new cases and 1.2 million deaths annually [2]. Over 700,000 survivors face permanent functional impairments, severely limiting societal or occupational reintegration [2]. Approximately 85% of strokes constitute ischemic stroke, presenting with transient or permanent neurological deficits [3]. It results from cerebral hypoperfusion due to arterial occlusion or embolism, causing tissue ischemia and subsequent neuronal necrosis [3]. Recanalization within the narrow therapeutic window via thrombolysis is a primary strategy. However, rapid reperfusion of blood flow provokes cerebral ischemia-reperfusion injury (CIRI) [4]. Due to this time constraint and medical technology limitations, only a small proportion of patients receive timely treatment [5, 6]. Therefore, a comprehensive understanding of the molecular mechanisms underlying CIRI is essential for developing intrinsic neuroprotective strategies.

Microglia, the resident immune cells of the central nervous system (CNS), play a crucial role in maintaining normal brain function and preserving the immune-privileged status [7]. Following ischemia, microglia rapidly activate and initiate the early immune response. During this process, they undergo structural, phenotypic, and functional transformations, exerting a dual role in both neural injury and repair. Proinflammatory cytokines, such as TNF-α, released by reactive microglia can directly induce neuronal death and disrupt blood-brain barrier (BBB) integrity [8, 9]. Activated microglia polarize into either an M1 phenotype (proinflammatory) or an M2 phenotype (anti-inflammatory) [10]. M1-type microglia promote cellular immune responses by producing inflammatory mediators such as IFN-β and IL-1β, contributing to neuronal damage and death. Conversely, M2-type microglia facilitate debris clearance, tissue repair, and secretion of anti-inflammatory cytokines, such as IL-10, IL-4, and TGF-β [11]. An increasing number of studies have highlighted the pivotal roles of microglia in CIRI. For instance, the Tat-NTS peptide protects against CIRI by modulating microglial polarization via induction of ANXA1 SUMOylation [12]. Similarly, downregulation of Nogo-B confers neuroprotection by regulating microglial polarization via inhibition of the TLR4/NF-κB signaling pathway [13]. ANXA1 has also been shown to promote a protective microglial phenotype through the FPR2/ALX-activated AMPK/mTOR pathway [14]. In addition, Qingda granule (QDG) attenuates neuroinflammation by suppressing the TLR4/NF-κB/NLRP3 axis in microglia [15]. Collectively, these findings suggest that shifting microglial polarization from the proinflammatory M1 phenotype toward the anti-inflammatory M2 phenotype may represent a promising therapeutic approach for CIRI, as the dynamic balance between these phenotypes plays a crucial role in neuronal activity and functional recovery [16]. However, the precise mechanistic role of microglia in CIRI remains incompletely elucidated.

PANoptosis, is a distinct programed cell death (PCD) pathway, involves the coordinated interplay of pyroptosis, apoptosis, and necroptosis beyond any single mechanism [17]. It is mediated by PANoptosome complexes integrating molecules from these pathways [17, 18], it plays critical roles in human diseases represents a promising therapeutic target [18–20]. Its significant involvement in ischemic stroke highlights therapeutic potential [21–23]. Vagus nerve stimulation inhibits PANoptosis via SIRT1, promoting recovery after cerebral ischemic stroke (CIS) [24]. Combined repeated transcranial magnetic stimulation (rTMS) and mesenchymal stem cell (MSC) transplantation inhibits neuronal PANoptosis and confers protection against CIRI [25]. Moreover, the neuronal LAMP2A-lysosomal pathway has been identified as a key inducer of a PANoptosis-like molecular cascade, driving acute neurodegenerative and neuroinflammatory responses following CIRI [26]. Additionally, inducing a shift of microglia toward the M2 phenotype represents a promising therapeutic strategy to attenuate neuronal PANoptosis in CIRI [27].

However, the effects of PANoptosis on diverse cell populations within the CIRI brain microenvironment and its associated biomarkers remain incompletely characterized.

In this study, we utilized single-cell RNA sequencing (scRNA-seq) and spatial transcriptomics (ST) data to identify the core ischemic region in stroke, referred to as the infarct core area (ICA), as well as the proximal (P) and distal (D) zones of the peri-infarct area (PIA). Furthermore, we investigated the expression patterns and functional roles of PANoptosis-related genes (PANRGs) in stroke, aiming to elucidate cell type-specific and region-specific apoptotic features. This comprehensive analysis may provide novel insights into potential therapeutic targets and strategies for stroke intervention and treatment.

## 2. Materials and Methods

### 2.1. Data Collection

scRNA-seq and spatial transcriptome RNA sequencing (stRNA-seq) data were obtained from the Genome Sequence Archive (GSA) (https://ngdc.cncb.ac.cn/gsa/) for four mice subjected to focal cortical ischemia via photothrombosis (PT) group and four mice with sham surgery (sham group), under accession numbers CRA006677 and CRA006678, respectively [28]. Additionally, bulk RNA-sequencing (bulk RNA-seq) data for ischemic stroke and sham mice were retrieved from the Gene Expression Omnibus (GEO) through the GSE137482 and GSE35338.

PANRGs were retrieved from the Molecular Signatures Database (MSigDB) (https://www.gsea-msigdb.org/gsea/msigdb) and a previous study [29], as shown in Supporting Information Table S1.

### 2.2. ScRNA-Seq Data Processing

“Seurat” R package was used to conduct the scRNA-seq analyses. To ensure data quality and integrity, genes expressed in at least three single cells were included in the analysis, while cells with fewer than 200 expressed genes, more than 7000 expressed genes, or over 20% mitochondrial and ribosomal gene expression were excluded. The “Harmony” package was used to correct for batch effects between groups [30]. The scRNA-seq data were then normalized using the “NormalizeData” function, and the top 2000 high-variance genes (HVGs) were identified using the “FindVariableFeatures” function. The “ScaleData” function was applied to normalize the expression matrix of HVGs. Principal component (PC) analysis (PCA) was performed using the “RunPCA” function based on these HVGs. Significant PCs were selected based on the proportion of variance explained. Subsequently, main clusters were identified using the “FindClusters” and “FindNeighbors” functions, and unbiased cell type identification was visualized using t-Distributed Stochastic Neighbor Embedding (t-SNE) [31]. The “FindAllMarkers” function was used to identify marker genes for each cluster via the Wilcoxon test. Cell populations were annotated based on marker genes and referenced from a previous study [28].

### 2.3. StRNA-Seq Data Processing

The “Seurat” R package was used to conduct the stRNA-seq analyses in this study. “FindVariableFeatures” was employed to identify 6000 HVGs, while standardization was performed using the “ScaleData” function. Normalization and scaling of unique molecular identifiers (UMIs) were carried out using the “SCTransform” function. Sample integration was performed after eliminating batch effects through canonical correlation analysis (CCA), using the “SelectIntegrationFeatures,” “FindIntegrationAnchors," and “IntegrateData” functions. Dimensionality reduction was performed using the “RunPCA” function. Cluster analysis was conducted using the “FindNeighbors” and “FindClusters” functions. Anatomical brain regions for each cluster were defined according to the Allen Mouse Brain Atlas (https://mouse.brain-map.org/static/atlas) and a previous study [28].

### 2.4. Multimodal Intersection Analysis (MIA) of Cell Type-Tissues Region Map

MIA was used to identify the cellular composition of distinct tissue regions and to determine the main cell types distributed within each spatial region [32]. Based on two gene sets from different sources specifically, the cell markers extracted from the scRNA-seq data and the genes corresponding to each cluster from ST-seq data, the MIA method was employed to explore the significant enrichment of the two gene sets. The enrichment significance was quantified as −log10 (phyper), where phyper represents the hypergeometric significance score, which is equivalent to −log10 (*p*-value). The regions showing the highest enrichment significance for intersections with specific cell types were considered as the major regions for those specific cell types.

### 2.5. Spatial Mapping of Cell Type Annotation

In the present study, “FindIntegationAnchors” function was used to discover cell-type-specific spatial patterns by mapping the annotated information from the scRNA-seq data to the stRNA-seq data as described in a previous study [28]. Cell type labels were transformed to ST data using “TransferData” function. Cell composition and distribution across different spatial regions were visualized using “SPOTlight” and “SpatialFeaturePlot” functions.

### 2.6. Cell-to-Cell Interaction Analyses

The “CellChat” package was used to explore the cell–cell interaction in both scRNA-seq data and stRNA-seq data [33, 34]. All the ligand–receptor (L–R) interactions were extracted from the Cell chartDB [33]. We comprehensively investigated the found types of cell–cell interactions, including autocrine, juxtacrine, paracrine, and endocrine [35]. The highly expressed genes (HEGs) between cell-types were identified using “identifyOverExpressedGenes” function, and interaction network of those HEGs were identified using the “identifyOverExpressedInteraction” function. The “computeCommunProb” and “computeCommunProbPathway” functions were used to infer the major incoming and outgoing signals across the signaling pathways. “AggregateNet” function was used to aggregate cell-to-cell communication network across different cell types. “getMaxWeight” function was used to predict the strength of interaction, and “contribution” function was used to infer the contributions to cell-to-cell communication.

### 2.7. Construction of Single-Cell Coexpression Networks

The “hdWGCNA” package was performed to construct the high-dimensional weighted gene coexpression network analysis (hdWGCNA) and to identify gene modules in both scRNA-seq data and stRNA-seq data [36]. Before constructing WGCNA, Seurat object data was constructed both in scRNA-seq and stRNA-seq data. Metacells in each group were constructed using “MetacellsByGroups” function, and the metacell expression matrix was normalized using “NormalizeMetacells” function. Subsequently, “TestSoftPowers” function was conducted to select optimal soft powers for constructing the adjacency matrix. The minimum soft power threshold was typically chosen to be the one with a scale-free topology model fit greater than or equal to 0.8. Module eigengenes and connectivity were calculated using “ScaleData” function, after which modules and hub genes were identified using the “GetModules” and “GetHubGenes” functions, respectively. The “ModuleExprscore” package was used to compute gene scores for the top 25 hub genes by intramodular connectivity (kME) for each module with UCell algorithm. The hub scores for each module were visualized using a feature plot by “ModulefeaturePlot” function.

### 2.8. Identification and Validation of Differentially Expressed PANRGs Using Bulk RNA-Seq

The differentially expressed genes (DEGs) between sham and stroke groups in GSE35338 dataset were identified suing “DESeq2” package with the absolute value of log2 (fold change [FC]) >0.5 and adjusted *p*-value (adj.*p*.Va) <0.05. Subsequently, the key different expressed PANRGs were obtained by intersecting DEGs from bulk RNA-seq data, DEGs from scRNA-seq data, DEGs from stRNA-seq data, and 120 PANRGs from the databases. Next, we explored the expression of the key genes between sham and stroke groups in GSE35338 and GSE137482 databases. Additionally, we also explored the expression of key PANRGs in single-cell and spatial region levels.

### 2.9. Gene Set Enrichment Analysis (GSEA)

To investigate the potential mechanisms of key PANRGs in stroke, Spearman correlation analysis was performed to evaluate the correlation between key PANRGs and all genes in the sham and stroke groups. Subsequently, KEGG enrichment analysis was conducted using the “clusterProfiler” package on these genes. Enriched signaling pathways were identified using the “GSEA” package with a *p*.adjusted <0.05 and visualized using the “GseaVis” package.

### 2.10. Construction of a Transcriptional Factor (TF)-microRNA (miRNA)-PANRGs Network

We next identify the TFs of key PANRGs using “RcisTarget” package. The upstream motifs of key PANRGs were extracted and subjected to normalized enrichment scoring (NES) and enrichment analysis using cumulative recovery curves. In the present study, we used the gene-motif rankings database for upstream and downstream 10 kb (mm10_refseq-r80_10kb_up_and_down_tss.mc9nr.genes_vs_motifs.rankings.feather, https://resources.aertslab.org/cistarget/databases/mus_musculus/mm10/refseq_r80/mc9nr/gene_based/) to predict transcription factor binding sites for key PANRGs.

Furthermore, mRNA–miRNA networks based on key PANRGs were constructed using miRWALK (http://mirwalk.umm.uni-heidelberg.de/) and miRDB (https://mirdb.org/), which are two websites for querying mRNA–miRNA relationships. The intersected miRNAs were obtained by intersecting the miRNAs from each website.

Finally, we constructed the TF-miRNA-key PANRGs regulatory network based on the top 50 TF and top 50 miRNAs, the network was visualized using Cytoscape software.

### 2.11. Gene Multiple Association Network Integration Algorithm (GeneMANIA)

GeneMANIA (https://genemania.org/) is a website used to explore the gene interactions and their gene functions [37]. “GOSemSim” package was conducted to explore the similarities among each key PANRG using gene ontology (GO)-terms Semantic Similarity Measures (GOSemSim) method [38].

### 2.12. Cell Culture and Oxygen-Glucose Deprivation/ReoxygenationOGD/R Model Establishment

Mouse BV2 microglial cell line (iCell-m011) was obtained from iCELL Bioscience Inc. (Shanghai, China) and cultured in Dulbecco's Modified Eagle Medium (DMEM) (Evacell, Suzhou, China) supplemented with 10% fetal bovine serum (FBS) (Evacell, Suzhou, China) and 1% penicillin–streptomycin (Evacell, Suzhou, China). This cell line was obtained from the German Collection of Microorganisms and Cell Cultures (DSMZ). It is derived from C57BL/6 mouse microglial cells and expresses nuclear v-myc and chromosomal v-raf oncogenes. The cells also express the surface envgp70 antigen and exhibit morphological, phenotypic, and functional characteristics typical of phagocytic cells. Cells were maintained under standard conditions at 37°C in a humidified atmosphere containing 95% air and 5% CO_2_.

To establish the OGD/R model, BV2 cells were first transferred to glucose-free DMEM (Procell, Wuhan, China; PM150270) and incubated at 37°C in a hypoxic chamber containing 5% CO_2_, 94% N_2_, and 1% O_2_ for 2 h. Subsequently, the cells were returned to normal glucose-containing DMEM and placed in a standard incubator to undergo reoxygenation for the indicated time periods (3, 6, 12, and 24 h).

### 2.13. Western Blot Analysis

Total protein lysates from BV2 microglial cells were extracted using RIPA lysis buffer (Beyotime, Shanghai, China) and incubated on ice for 30 min. Protein concentrations were determined using a bicinchoninic acid (BCA) protein assay kit (Beyotime, Shanghai, China). Equal amounts of protein samples were separated by electrophoresis on 4%–15% SDS-polyacrylamide gels and subsequently transferred onto PVDF membranes (Millipore, Billerica, MA, USA). Membranes were blocked with 5% skim milk in Tris-buffered saline with 0.1% Tween-20 (TBST) for 2–3 h at room temperature, followed by overnight incubation at 4 °C with a mouse anti-*TNFRSF1A* antibody (1:1000; ab223352, Abcam, Cambridge, UK). After three washes with TBST, membranes were incubated with the appropriate HRP-conjugated secondary antibody at room temperature for 1–2 h. Protein bands were visualized using enhanced chemiluminescence (ECL) reagents (Affinity, Liyang, China). β-actin was used as the internal loading control. Band intensities were quantified using ImageJ software.

## 3. Results

### 3.1. Identification of Nine Major Cell Populations and 14 Brain Regions in Mouse Brain Sections

**Figure 1:**
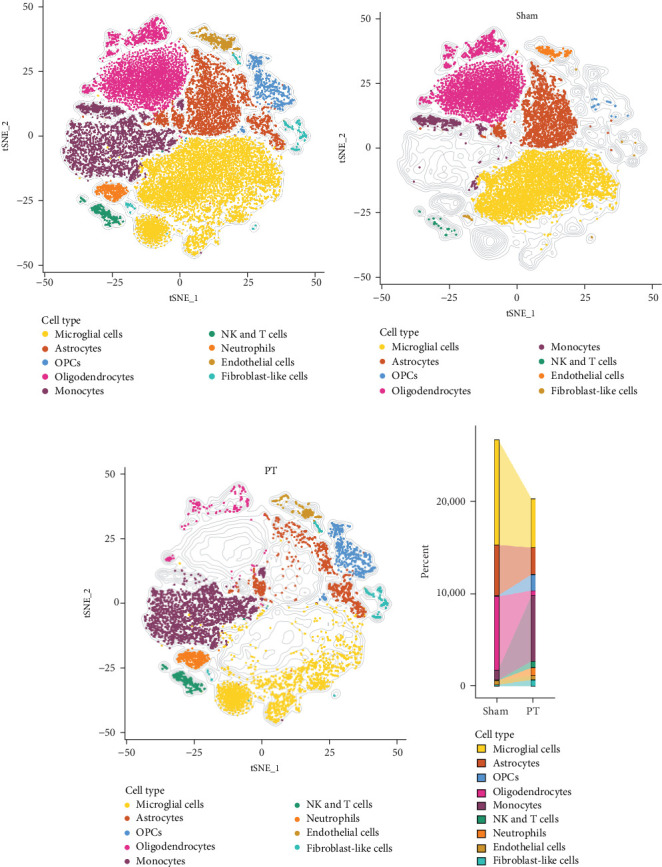
Identification of nine major cell populations in mouse brain sections. (A) T-SNE plots depicting the distribution of different cell types in mouse brain sections. (B) T-SNE plots depicting the distribution of different cell types in mouse brain sections from the sham surgery group (sham group). (C) T-SNE plots depicting the distribution of different cell types in mouse brain sections following focal cortical ischemia induced by photothrombosis (PT group). (D) Histogram illustrating the distribution of cell types across different experimental groups.

We analyzed a publicly available scRNA-seq dataset from PT *n* = 4) and sham (*n* = 4) mouse brains. After quality control (Supporting Information Figure S1A–G), 47,070 cells were retained and classified into 13 clusters corresponding to nine major cell types (Figure 1A, Supporting Information Figure S1H–O). Compared with the sham group, PT brains showed increased monocytes and fibroblast-like cells but reduced microglia, astrocytes, and oligodendrocytes (Figure 1B–D). These findings highlight the cellular heterogeneity in the mouse brain after focal cortical ischemia.

stRNA analysis further mapped 25 niches to 14 brain regions, with the ICA andPIA_P and PIA_D showing the most pronounced changes (Supporting Information Figures S2A–B, S3A–B, and S4A–B). MIA revealed strong associations between microglia and ICA, and monocytes and PIA_P (Supporting Information Figure S4C–E,G). Oligodendrocytes and OPCs were enriched in the isocortex and white matter of sham mice, whereas microglia accumulated in ICA and astrocytes and monocytes in PIA_P of PT mice (Supporting Information Figure S4F,H), suggesting that regional microglial enrichment in ICA may contribute to tissue damage following ischemia.

### 3.2. Intercellular Communications in the Mouse Brain Following Focal Cortical Ischemia

**Figure 2:**
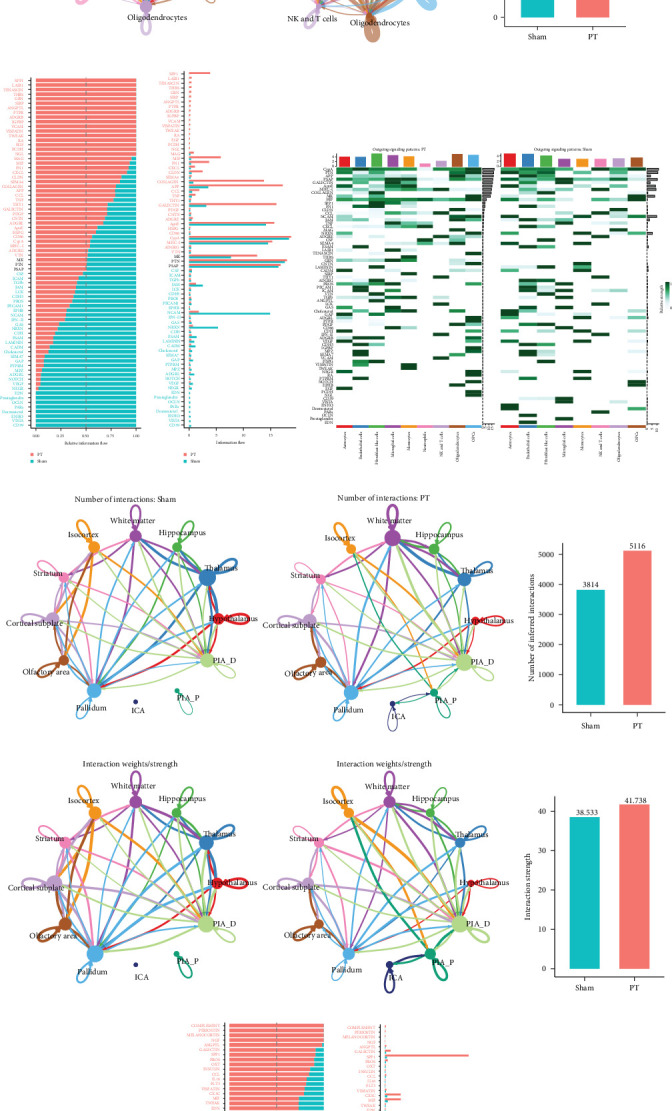
Intercellular communications in the mouse brain following focal cortical ischemia. (A) Overall interaction networks showing the total number of interactions across nine cell types in the sham and PT groups of scRNA-seq data. (B) Histogram illustrating the differences in the number of interactions between the sham and PT groups in scRNA-seq data. (C) Interaction networks depicting the predicted interaction strength across nine cell types in the sham and PT groups of scRNA-seq data. (D) Histogram illustrating the differences in interaction strength between the sham and PT groups in scRNA-seq data. (E) Histogram illustrating the overall information flow for each signaling pathway in scRNA-seq data. (F) Histogram showing the differentially significant signaling pathways between the sham and PT groups in scRNA-seq data. (G) Heatmap displaying the outgoing signaling patterns in the sham and PT groups. (H) Overall interaction networks showing the total number of interactions across nine cell types in the sham and PT groups of stRNA-seq data. (I) Histogram illustrating the differences in the number of interactions between the sham and PT groups in stRNA-seq data. (J) Interaction networks depicting the predicted interaction strength across nine cell types in the sham and PT groups of stRNA-seq data. (K) Histogram illustrating the differences in interaction strength between the sham and PT groups in stRNA-seq data. (L) Histogram illustrating the overall information flow for each signaling pathway in stRNA-seq data. (M) Histogram showing the differentially significant signaling pathways between the sham and PT groups in stRNA-seq data.

Given the cellular heterogeneity in the mouse brain after focal cortical ischemia, we hypothesized that intercellular communications among different cell types are critical for pathological progression. Compared with the sham group, the PT group exhibited a higher overall number and strength of interactions (Figure 2A–D). In particular, activation interactions were observed among microglia, astrocytes, oligodendrocytes, and OPCs. At the single-cell level, L–R analysis revealed 76 signaling pathways across nine cell types (Figure 2E–G). Pathways significantly enriched in the PT group included phosphoprotein 1 (SPP1), macrophage migration inhibitory factor (MIF), fibronectin (FN1), collagen, amyloid precursor protein (APP), galectin, CC motif chemokine ligand (CCL), CXC chemokine ligand (CXCL), and tumor necrosis factor (TNF), suggesting stronger inflammatory responses, fibrosis, tissue remodeling, and nerve damage/repair (Figure 2E–G). ST further confirmed these findings, revealing increased interactions and enrichment of galectin, SPP1, CX3C, MIF, and CSF signaling in the PT group (Figure 2H–M). Regionally, MIF-CD74, MIF-CD44, and MIF-CXCR4 were highly expressed in ICA, PIA_P, and PIA_D areas (Supporting Information Figure S5A), while LGALS9-LGHM and LGALS9-CD44 were similarly enriched (Supporting Information Figure S5B).

**Figure 3:**
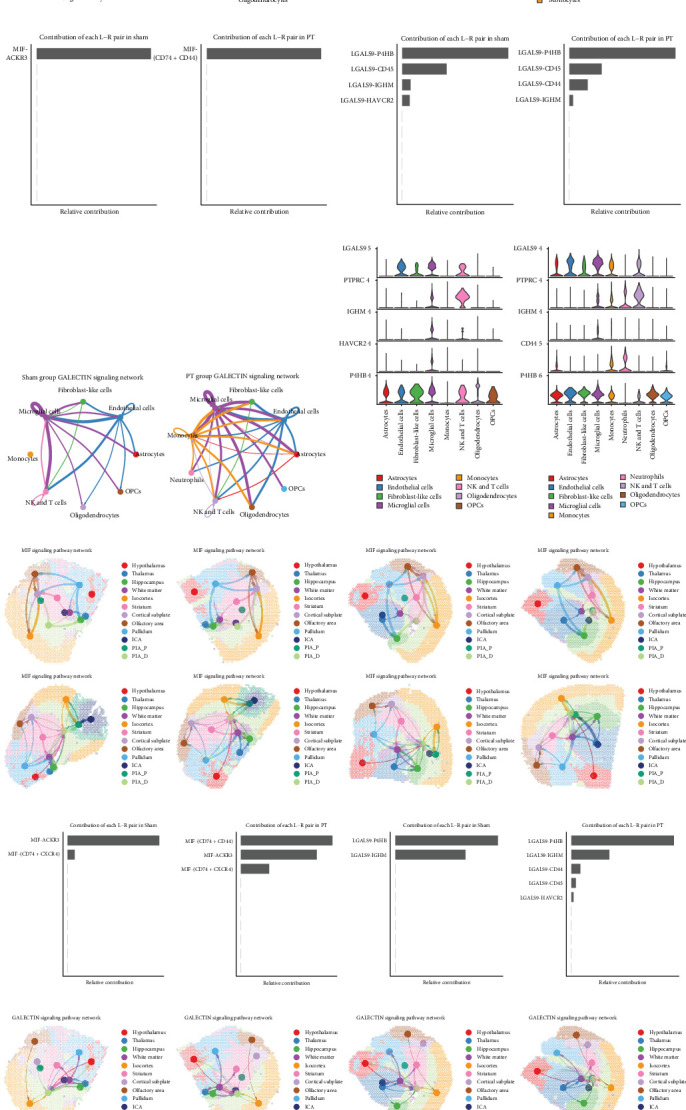
Significant pathways in intercellular communications in the mouse brain following focal cortical ischemia. (A) Network depicting the predicted interaction strength of the MIF ligand–receptor pair in the sham and PT groups. (B) Violin plot showing the expression of the MIF ligand–receptor pair across different cell types in the sham and PT groups. (C) Histogram illustrating the contributions of the MIF ligand–receptor pair in various cell types in the sham and PT groups. (D) Histogram illustrating the contributions of the LGALS ligand–receptor pair in different cell types in the sham and PT groups. (E) Network depicting the predicted interaction strength of the LGALS ligand–receptor pair in the sham and PT groups. (F) Violin plot showing the expression of the LGALS ligand–receptor pair across different cell types in the sham and PT groups. (G) Network illustrating the information flow of the MIF ligand–receptor pair in the sham and PT groups. (H) Histogram displaying the contributions of the MIF ligand–receptor pair in different spatial regions in the sham and PT groups. (I) Histogram displaying the contributions of the LGALS ligand–receptor pair in different spatial regions in the sham and PT groups. (J) Network illustrating the information flow of the LGALS ligand–receptor pair in the sham and PT groups.

Integrated analysis of scRNA-seq and stRNA-seq data highlighted GALECTIN and MIF signaling as key pathways in ischemic pathology. Specifically, immune-related genes were differentially expressed in various cell types via MIF signaling (Figure 3A). For example, MIF-ACKR3 was enriched in fibroblasts of the sham group, while MIF-CD74 was upregulated in endothelial cells, fibroblasts, microglia, and monocytes; MIF-CD44 was highly expressed in astrocytes, monocytes, and neutrophils in the PT group (Figure 3B–D). Similarly, GALECTIN signaling involved multiple L–R pairs broadly expressed across cell types (Figure 3E), consistent with previous reports [28]. LGALS9-P4HB was predominant in ECs, fibroblasts, microglia, and NK and T cells in the sham group, whereas in the PT group, LGALS9-CD45, LGALS9-LGHM, and LGALS9-CD44 were enriched in microglia, monocytes, neutrophils, and NK and T cells, with LGALS9-P4HB broadly expressed in astrocytes, ECs, fibroblasts, and immune cells (Figure 3F). Collectively, these results demonstrate that GALECTIN and MIF signaling pathways orchestrate intercellular communication and contribute centrally to the pathological responses following focal cortical ischemia.

### 3.3. Identification of Hub Genes Using hdWGCNA Both in Single-Cell and Spatial Levels

**Figure 4:**
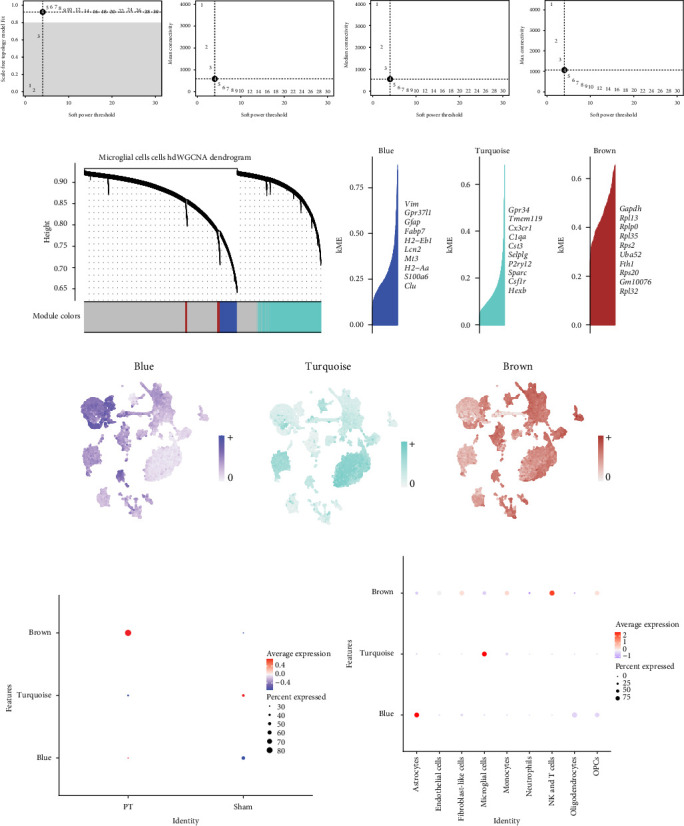
Identification of hub genes using hdWGCNA by scRNA-seq data. (A) Scale-free topology model fit index and mean connectivity for various soft-thresholding powers. (B) Dendrogram displaying the hierarchical clustering of microglial cell genes based on their expression patterns, as determined by weighted gene coexpression network analysis (WGCNA). (C) Eigengene-based connectivity (kME) map showing the top 10 hub genes in each module, ranked by kME across microglial cells. (D) T-SNE plots illustrating the expression distribution of hub genes for each module across nine different cell types. (E) Dot plots depicting the average expression of module-specific hub genes in the sham and PT groups, based on gene scores calculated by the UCell algorithm for the top 25 hub genes by kME in each module. (F) Dot plots showing the average expression of module-specific hub genes across different cell types, based on gene scores calculated by the UCell algorithm for the top 25 hub genes by kME in each module.

Given the pivotal roles of microglial cells after focal cortical ischemia, we first applied hdWGCNA to construct a coexpression network (soft power = 4), which identified four gene modules (Figure 4A,B). Among them, three gene modules (blue, turquoise, and brown) were selected for hub gene analysis (Figure 4C). UCell scoring of the top 25 hub genes per module revealed cell type-specific expression (Figure 4D). Compared with the sham group, gene scores of the brown and turquoise modules were elevated, whereas those of the blue module were reduced (Figure 4E). Notably, the brown and turquoise modules were enriched in microglia and NK and T cells, while the blue module was enriched in astrocytes (Figure 4F). In total, 2710 module genes were extracted for further analysis (Supporting Infromation Table S2).

**Figure 5:**
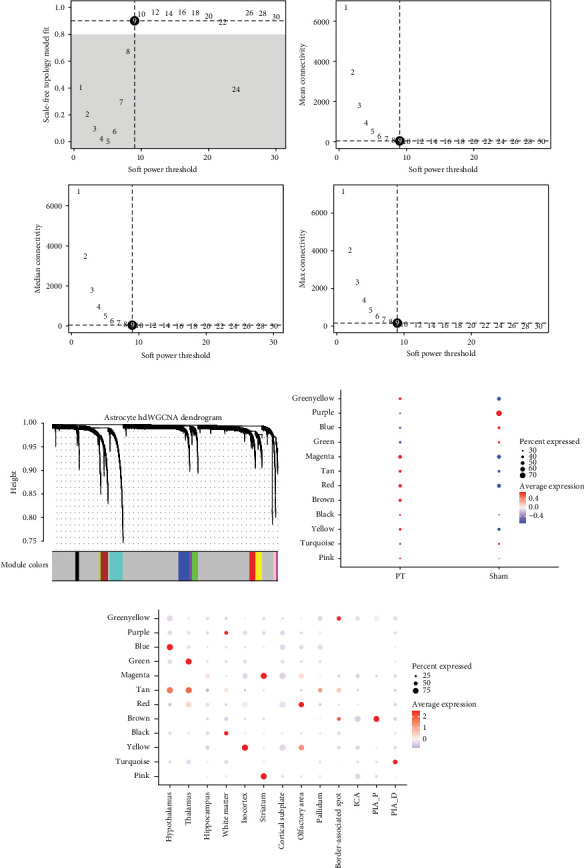
Identification of hub genes using hdWGCNA by stRNA-seq data. (A) Scale-free topology model fit index and mean connectivity for various soft-thresholding powers. (B) Dendrogram displaying the hierarchical clustering of astrocyte genes based on their expression patterns, as determined by weighted gene coexpression network analysis (WGCNA). (C) Dot plots depicting the average expression of module-specific hub genes in the sham and PT groups, based on gene scores calculated by the UCell algorithm for the top 25 hub genes by kME in each module. (D) Dot plots showing the average expression of module-specific hub genes across different cell types, based on gene scores calculated by the UCell algorithm for the top 25 hub genes by kME in each module.

Similarly, considering the importance of astrocytes, we constructed an hdWGCNA network (soft power = 9), identifying 12 distinct modules (Figure 5A,B). UCell analysis showed differential hub gene expression across spatial niches (Supporting Information Figure S6A–B). Correlation analysis revealed that eight modules (greenyellow, magenta, tan, red, brown, black, yellow, and pink) were elevated, while four modules (purple, blue, green, and turquoise) were decreased in the PT group (Figure 5C). Module–niche associations were observed, including blue/tan with the hypothalamus, green/tan with the thalamus, purple/black with the hippocampus, yellow with the isocortex, magenta/pink with the striatum, red with the olfactory area, greenyellow with the border-associated spot, brown with PIA_P, and turquoise with PIA_D (Figure 5D). In total, 3579 module genes were extracted for further investigation (Supporting Information Table S3).

### 3.4. Identification of the Hub PANRGs in Stroke Mice

**Figure 6:**
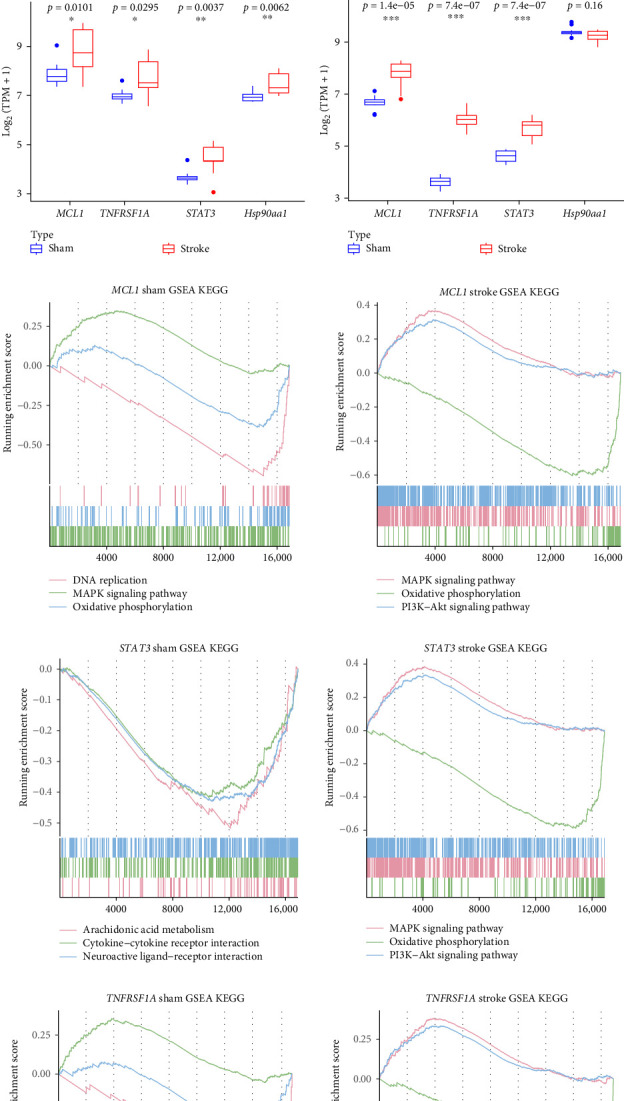
Identification of the hub PANRGs in stroke mice. (A) Volcano plot depicting the differentially expressed genes (DEGs) between stroke and sham groups in the GSE35338 dataset, with thresholds of |log_2_ FC| > 0.5 and adj.*p*-value < 0.05. (B) Venn diagram illustrating the key differentially expressed PANRGs by intersecting DEGs from bulk RNA-seq, scRNA-seq, and stRNA-seq data with PANRGs from relevant databases. (C,D) Boxplots showing the expression levels of *MCL1*, *TNFRSF1A*, *STAT3*, and *Hsp90aa1* between stroke and sham groups in the GSE35338 and GSE137482 datasets. (E–G) GSEA plots displaying significantly enriched pathways in the sham and PT groups.

Considering that TNF, SPP1, MIF, and GALECTIN signaling pathways are involved in various forms of programed cell death (PCD) [7–10], we hypothesized that PANoptosis contributes critically to ischemic stroke pathology. We therefore sought to identify key PAN-regulated genes (PANRGs) in stroke. From the GSE35338 dataset, we identified 1711 DEGs (1143 upregulated and 568 downregulated) between stroke and sham groups (Figure 6A, Supporting Information Table S4). Intersecting 120 PANRGs with scRNA-seq, stRNA-seq, and bulk RNA-seq data yielded four hub PANRGs, including *MCL1*, *TNFRSF1A*, *STAT3*, and *Hsp90aa1* (Figure 6B). Validation in GSE35338 and GSE137482 showed consistent upregulation of *MCL1*, *TNFRSF1A*, and *STAT3* in stroke (Figure 6C,D), supporting their role as candidate biomarkers. Functional analysis indicated that these genes participate in cellular signaling, metabolism, and energy generation-related pathways, including DNA replication, nucleotide excision repair, fatty acid degradation, and oxidative phosphorylation (Supporting Information Figure S7A–C). Notably, their high expression correlated with activation of MAPK and PI3K-AKT pathways (Figure 6E,F) and suppression of oxidative phosphorylation (Figure 6G). Collectively, these findings suggest that *MCL1*, *TNFRSF1A*, and *STAT3* regulate PANoptosis and contribute to stroke progression.

### 3.5. Analysis of the Hub PANRGs Expression in Single-Cell and Spatial Levels

**Figure 7:**
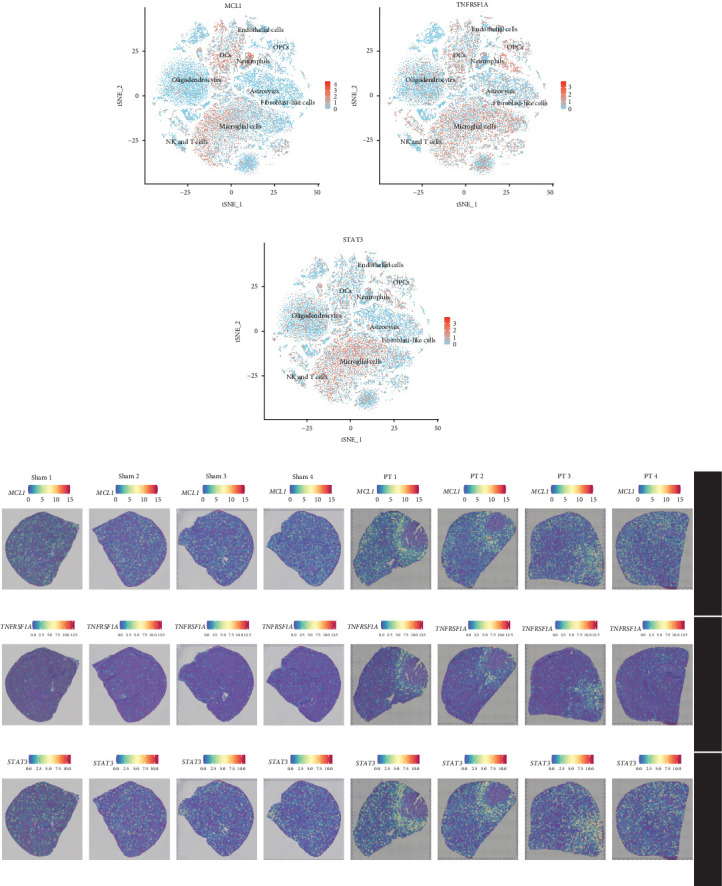
Analysis of the hub PANRGs expression at single-cell and spatial levels. (A–C) T-SNE plots showing the expression distribution of hub genes (*MCL1*, *TNFRSF1A*, *STAT3*) across nine distinct cell types. (D–F) Expression distribution of hub genes (*MCL1*, *TNFRSF1A*, *STAT3*) across different spatial regions in the sham and PT groups.

At both single-cell and spatial levels, *MCL1*, *TNFRSF1A*, and *STAT3* were significantly expressed in microglial cells (Figure 7A–C) and enriched in the PIA_P spatial region (Figure 7D–F). Further analysis showed activation of the TNF signaling pathway through the TNF-*TNFRSF1A* interaction in the PT group (Supporting Information Figure S8A). Specifically, the TNF-*TNFRSF1A* pair was highly expressed in microglial cells and NK and T cells in the sham group, but extended to fibroblasts, microglia, monocytes, and OPCs in the PT group (Supporting Information Figure S8B,C). These findings highlight *TNFRSF1A* plays as a key mediator of intercellular communication and histopathological processes in stroke.

### 3.6. Construction of a TF-miRNA-PANRGs Network and GeneMANIA Analysis

**Figure 8:**
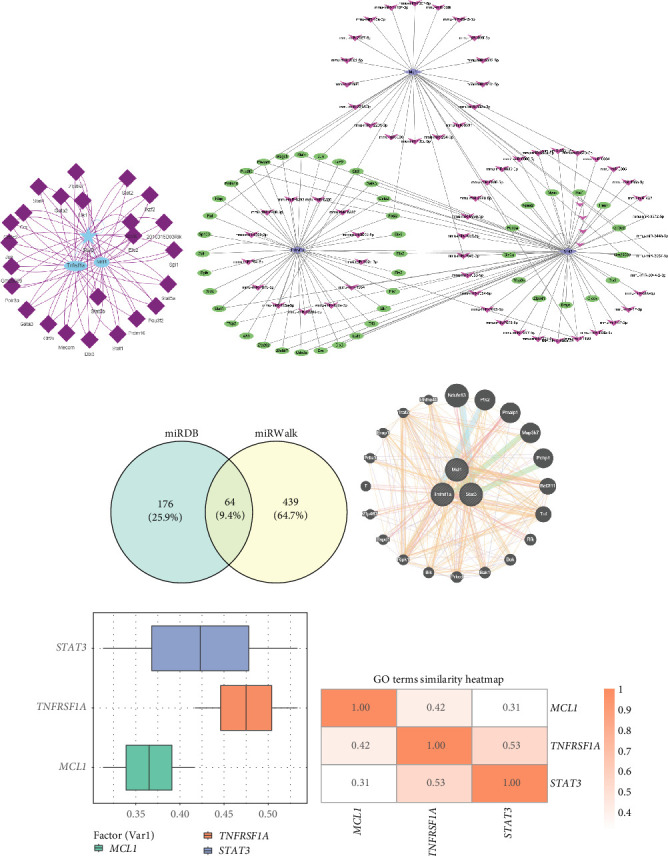
Construction of a TF-miRNA-PANRGs network and GeneMANIA. (A) Network depicting the top 25 transcription factors (TFs) associated with *MCL1*, *TNFRSF1A*, and *STAT*3. (B) Network illustrating the TF-miRNA-PANRGs interactions based on the top 50 TFs, top 50 miRNAs, and *MCL1*, *TNFRSF1A*, and *STAT3*. (C) Venn diagrams showing the intersecting miRNAs targeting *MCL1*, *TNFRSF1A*, and *STAT3*, as identified from the miRWalk and miRDB databases. (D) Network displaying the functionally similar genes predicted by GeneMANIA. (E) Heatmap showing the biomarker functional similarity. (F) Boxplot displaying the biomarker functional similarity.

To investigate the regulatory mechanisms of *MCL1*, *TNFRSF1A*, and *STAT3*, we identified transcription factors (TFs) through enrichment analysis using cumulative recovery curves (Supporting Information Figure S9A–E). The top 25 TFs associated with *MCL1*, *TNFRSF1A*, and *STAT3* were then selected (Figure 8A). Since miRNAs regulate gene expression by binding to mRNAs, we further screened potential candidates using miRWalk and miRDB databases, we identified 64 miRNAs that target all three PANRGs (Figure 8C, Supporting Information Tables S5, S6 and S7). Based on these results, we constructed a TF-miRNA-PANRGs network comprising the top 50 TFs, 50 miRNAs, and the three hub genes (Figure 8B). We next applied GeneMANIA to build a protein–protein interaction (PPI) network for genes functionally related to *MCL1*, *TNFRSF1A*, and *STAT3* (Figure 8D). These associated genes were enriched in pathways linked to cell death (apoptosis and necrosis), protease regulation, signal transduction, and mitochondrial function (Supporting Information Table S8). Functional similarity analysis further confirmed close associations among the three hub genes (Figure 8E,F). Collectively, these findings suggest that the TF-miRNA-PANRG regulatory network, together with functionally related genes identified by GeneMANIA, provides new insights into the molecular mechanisms of PANoptosis in ischemic stroke.

### 3.7. The Expression of *TNFRSF1A* is Increased After CIRI

**Figure 9:**
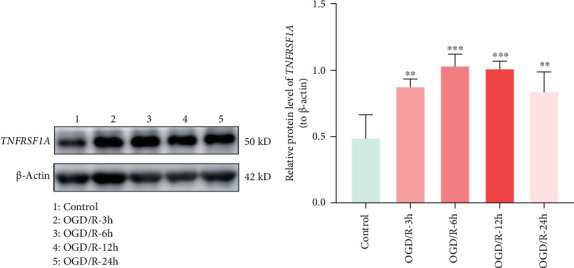
The expression of *TNFRSF1A* after CIRI. (A) The images of *TNFRSF1A* and β-actin at different timepoints. (B) The expression of *TNFRSF1A* at different timepoints. *⁣*^*∗∗*^*p* < 0.01 and *⁣*^*∗∗∗*^*p* < 0.001.

The protein levels of *TNFRSF1A* were evaluated by Western blot analysis at various time-points following CIRI. Compared with the control group, *TNFRSF1A* expression was significantly upregulated after CIRI, reaching its peak at 6 h postinjury (Figure 9A,B). These results suggest that *TNFRSF1A* may play a key role in the acute phase of CIRI injury.

## 4. Discussion

In this study, we combined ST and scRNA-seq with MIA to map cell population enrichment following stroke, providing a detailed atlas of diverse cell types and subsets. Microglial cells emerged as a key population in the poststroke environment, supporting the rationale for targeting specific microglial subclusters as the potential therapeutic strategies [11]. Consistent with our findings, Han et al. [28] identified LGALS9 as a therapeutic target in the PIA of a murine stroke model. ST analysis revealed global spatial and transcriptional remodeling in the ischemic hemisphere. By clustering tissue spots based on spatially variable gene expression, we identified distinct ischemic regions, including the ischemic core area (ICA), PIA_P, and PIA_D. This unbiased approach delineated peri-infarct regions and highlighted transcriptional differences between P and D zones, reflecting progressive cortical reorganization, particularly in primary and secondary motor cortices.

Notably, the ICA region showed a strong association with microglial cells, evidenced by the presence of three distinct microglial biomarkers. Integration with scRNA-seq allowed refined spatial annotation of ischemia-related gene expression. Analysis of intercellular communication revealed that the LGALS9-LGHM and LGALS9-CD44 L–R) pairs were highly expressed in the ICA, PIA_P, and PIA_D regions in the PT stroke group. Galectin-9 (LGALS9), primarily originating from microglia and macrophages, is known for immune regulation in tumors [16] and has been associated with large artery atherosclerotic stroke [39], highlighting its potential role in postischemic inflammation [28].

Microglia play a critical role in neuroinflammation following ischemic injury [10]. Previous studies have shown that microglial activation exhibits distinct spatial and temporal patterns during the course of ischemic injury [40, 41]. Importantly, microglial responses are shaped by polarization dynamics, and modulating microglial polarization has emerged as a promising therapeutic strategy. Although our study did not explicitly distinguish between different polarized microglial subtypes, we observed that microglial cells were highly active in the infarcted area. Notably, increased LGALS9 and CD44 expression has been linked to inflammatory activation [42, 43]. The spatial enrichment of LGALS9-CD44 signaling in the ICA region suggests that this pathway may regulate microglial polarization, thereby modulating local inflammatory responses and potentially priming cells for PANoptotic cell death pathways in ischemic regions.

Elevated levels of PANoptosis-associated proteins have been observed in retinal neurons in response to ischemia/reperfusion (I/R) injury [44]. We further investigated PANoptosis-associated genes in stroke. Four hub PANRGs (*MCL1*, *TNFRSF1A*, *STAT3*, *Hsp90aa*1) were identified, enriched in MAPK and PI3K-AKT signaling pathways and negatively associated with oxidative phosphorylation, suggesting their involvement in stroke pathogenesis. Notably, *TNFRSF1A* was significantly expressed in microglial cells within the PIA_P at both single-cell and ST levels. Furthermore, experimental analyses confirmed that the protein level of *TNFRSF1A* was markedly upregulated following CIRI. *TNFRSF1A* gene encodes tumor necrosis factor receptor 1 (*TNFRSF1A*) and has been implicated in poststroke depression [45], where cerebellar fastigial nucleus stimulation reduces its expression to attenuate inflammation and disease severity [46]. It is also linked to reduced kidney function in ischemic heart disease and diabetes [47, 48], and its expression correlates with neuroinflammatory responses across multiple neurological disorders [49–52]. Collectively, these findings suggest that *TNFRSF1A* may serve as a promising therapeutic target in stroke.

Importantly, these observations raise the possibility that LGALS9-CD44 signaling may interact with *TNFRSF1A*-mediated pathways to modulate PANoptosis in microglia. *TNFRSF1A*, a key receptor in TNF signaling, has been implicated in the activation of downstream MAPK and PI3K-AKT pathways [53–55], which are critical regulators of PANoptotic cell death. Han et al. [28] have demonstrated that LGALS9-CD44 is primarily secreted by microglial cells and plays a critical role following ischemic injury. We speculated that the upregulation of LGALS9-CD44 in ischemic regions may facilitate paracrine or juxtacrine signaling that primes microglia for *TNFRSF1A*-dependent PANoptotic execution. This potential crosstalk between intercellular communication pathways and programed cell death (PCD) mechanisms could help explain the enhanced inflammatory and cell death responses observed in the ICA, PIA_P, and PIA_D regions following stroke, linking spatially resolved L–R interactions to core molecular processes driving tissue damage. To validate these findings, we established an oxygen-glucose deprivation/reoxygenation (OGD/R) model using BV2 cells. We observed that *TNFRSF1A* expression was significantly upregulated after CIRI in a time-dependent manner, suggesting a critical role for *TNFRSF1A* during the acute phase of injury. This result aligns with previous studies reporting increased *TNFRSF1A* expression in OGD/R-induced injury models [56]. Additionally, *TNFRSF1A* upregulation has been associated with caspase-8- and gasdermin D (GSDMD)-dependent PANoptosis in the seasonal atrophy of scented glands in male muskrats [57], and *TNFRSF1A* overexpression correlates with poor prognosis across multiple cancer types and participates in PANoptosis in nonalcoholic fatty liver disease (NAFLD) [58]. Together, these findings highlight that *TNFRSF1A* may drive PANoptosis and contribute to disease progression.

While our study is the first to identify hub PANRGs in stroke and elucidate their associated cell types and brain region, certain limitations remain. The relatively small sample size may limit the generalizability of our findings, and the precise mechanisms by which *TNFRSF1A* contributes to stroke pathophysiology are still unclear. Notably, neurons were largely absent in our single-cell dataset after quality control, which may be due to factors such as tissue dissociation bias, the postinjury stage at which samples were collected, and quality control (QC) thresholds that disproportionately filter neuronal cells. Comparable studies have detected neuronal clusters in ischemic-brain single-cell data [59, 60], highlighting differences that may arise from experimental protocols or injury timing. Moreover, our dataset samples were collected 3 days after model induction and were not stratified by different time points (e.g., hyperacute or acute phases), making it impossible to analyze the temporal dynamics of microglia following ischemic injury or their relationship with clinical stages. Future investigations with larger cohorts, additional time points, and mechanistic studies in vitro and in vivo are warranted to validate these results and to explore the therapeutic potential of *TNFRSF1A* in ischemic stroke.

## 5. Conclusion

By integrating scRNA-seq and ST analyses, this study provides a comprehensive spatial landscape of distinct cell subpopulations following stroke. Our results demonstrate that *TNFRSF1A* is highly expressed in microglial cells and specifically upregulated in ischemic regions, highlighting its potential role in PANoptosis. These findings suggest that *TNFRSF1A* may serve as a novel prognostic biomarker and a promising therapeutic target for ischemic stroke.

## Data Availability

All data generated or analyzed during this study are included in this published article and its supporting information files.

## Supporting Information

Supporting Information 1 Table S1. PANoptosis-related genes (PANRGs) were retrieved from the MSigDB and article.

Supporting Information 2 Table S2. The scDEGs from the hdWGCNA based on scRNA-seq data.

Supporting Information 3 Table S3. The stDEGs from the hdWGCNA based on stRNA-seq data.

Supporting Information 4 Table S4. The differentially expressed genes (DEGs) between the stroke and sham groups in the GSE35338 dataset.

Supporting Information 5 Table S5. miRNAs identified from the miRWalk.

Supporting Information 6 Table S6. miRNAs identified from the miRDB.

Supporting Information 7 Table S7. The intesected miRNAs identified from the miRWalk and miRDB.

Supporting Information 8 Table S8. The pathways predicted in GeneMANIA database.

Supporting Information 9 Figure S1. Quality control, normalization, clustering of scRNA-seq data, and cell annotation. (A–D) Violin plots displaying the number of RNA features (nFeature_RNA), absolute UMI counts (nCount_RNA), and percentage of mitochondrial genes (mt_percent) before and after quality control filtering of each sequencing cell in the sham and PT groups. (E) Histogram showing the distribution of unique genes and transcripts per cell. (F–G) Dimensionality reduction plots displaying the results before and after Harmony integration of each sequencing cell in the sham and PT groups. (H) Volcano plots showing the high variable genes (HVGs). (I) Elbow plots of principal component analysis (PCA). (J) UMAP plots showing the different clusters at varying resolutions. (K–M) T-SNE plots showing the 13 clusters at a resolution of 0.1. (M) Bubble plots displaying the top two marker genes for each cell type. (N) Heatmap showing the top five marker genes for each cell type.

Supporting Information 10 Figure S2. Normalization of stRNA-seq data. (A, B) Normalization and integration of the stRNA-seq data using canonical correlation analysis (CCA) in the sham and PT groups.

Supporting Information 11 Figure S3. Clustering of stRNA-seq data. (A, B) Dimensionality reduction by PCA.

Supporting Information 12 Figure S4. Annotation of the niches in stRNA-seq data, and identification of fourteen brain regions and specific cell types in each region. (A,B) Clustering of 25 niches using 30 principal components (PCs) and a resolution of 0.8 in the sham and PT groups. (C,D) Violin plots displaying the UMI counts per spot and genes per spot in different regions. (E) H and E staining of mouse brain sections from the sham (*n* = 4) and PT (*n* = 4) groups, with transcriptome sections. (F) Spot clustering and annotation in the stRNA-seq data. (G) MIA heatmap showing the correlation significance analysis between stRNA-seq data and cell subsets from scRNA-seq data. −Log_10_ (phyper) is equivalent to −log_10_ (*p*-value). (H) Mapping of cell types from scRNA-seq data to stRNA-seq data using the “FindIntegrationAnchors” function.

Supporting Information 13 Figure S5. Expression of ligand–receptor pairs across different spatial regions. (A) Expression of the MIF-ACKR3, MIF-CD74, MIF-CD44, and MIF-CXCR4 pairs in different spatial regions in the sham and PT groups. (B) Expression of the LGALS9-P4HB, LGALS9-LGHM, LGALS9-CD44, and LGALS9-HAVCR2 pairs in different spatial regions in the sham and PT groups.

Supporting Information 14 Figure S6. Expression of module-specific hub genes across different spatial regions. (A, B) The average expression of module-specific hub genes across different spatial regions in the sham and PT groups, based on gene scores calculated by the UCell algorithm for the top 25 hub genes by kME in each module.

Supporting Information 15 Figure S7. Gene Set Enrichment Analysis (GSEA). (A) The top 30 enriched pathways for *MCL1* in the stroke and sham groups. (B) The top 30 enriched pathways for *STAT3* in the stroke and sham groups. (C) The top 30 enriched pathways for *TNFRSF1A* in the stroke and sham groups. Red represents the *p*-value, and the size of the dot represents the number of enriched genes.

Supporting Information 16 Figure S8. TNF significant pathways in intercellular communications in the mouse brain following focal cortical ischemia. (A) Network displaying the predicted interaction strength of the TNF ligand–receptor pair in the sham and PT groups. (B) Violin plot showing the expression of the TNF ligand–receptor pair across different cell types in the sham and PT groups. (C) Histogram illustrating the contributions of the TNF ligand–receptor pair in various cell types in the sham and PT groups.

Supporting Information 17 Figure S9. Identification of the transcription factors (TFs) of *MCL1*, *TNFRSF1A*, and *STAT3* using the “RcisTarget” package. (A) Global mean and standard deviation estimation. Shows the mean and standard deviation estimates for global gene recovery. (B) Enrichment analysis of the motif dbcorrdb__STAT3__ENCSR000DOQ_1__m1 in the gene set. (C) Enrichment analysis of the motif dbcorrdb__STAT3__ENCSR000DOX_1__m1 in the gene set. (D) Enrichment analysis of the motif taipale_tf_pairs__ETV2_DLX3_RSCGGAANNNNNNYAATTA_CAP in the gene set. (E) The top ten enriched motifs for *MCL1*, *TNFRSF1A*, and *STAT3* and their corresponding transcription factors (TFs).
