# Supplementary figures and images for: Comprehensive Analysis of the PANoptosis-Related Genes in Stroke Based on Single-Cell RNA-Seq and Spatial Transcriptomics
Source: Mediators Inflamm. 2025 Nov 4;2025:5828665. doi: 10.1155/mi/5828665 (PMC12605869; doi:10.1155/mi/5828665)

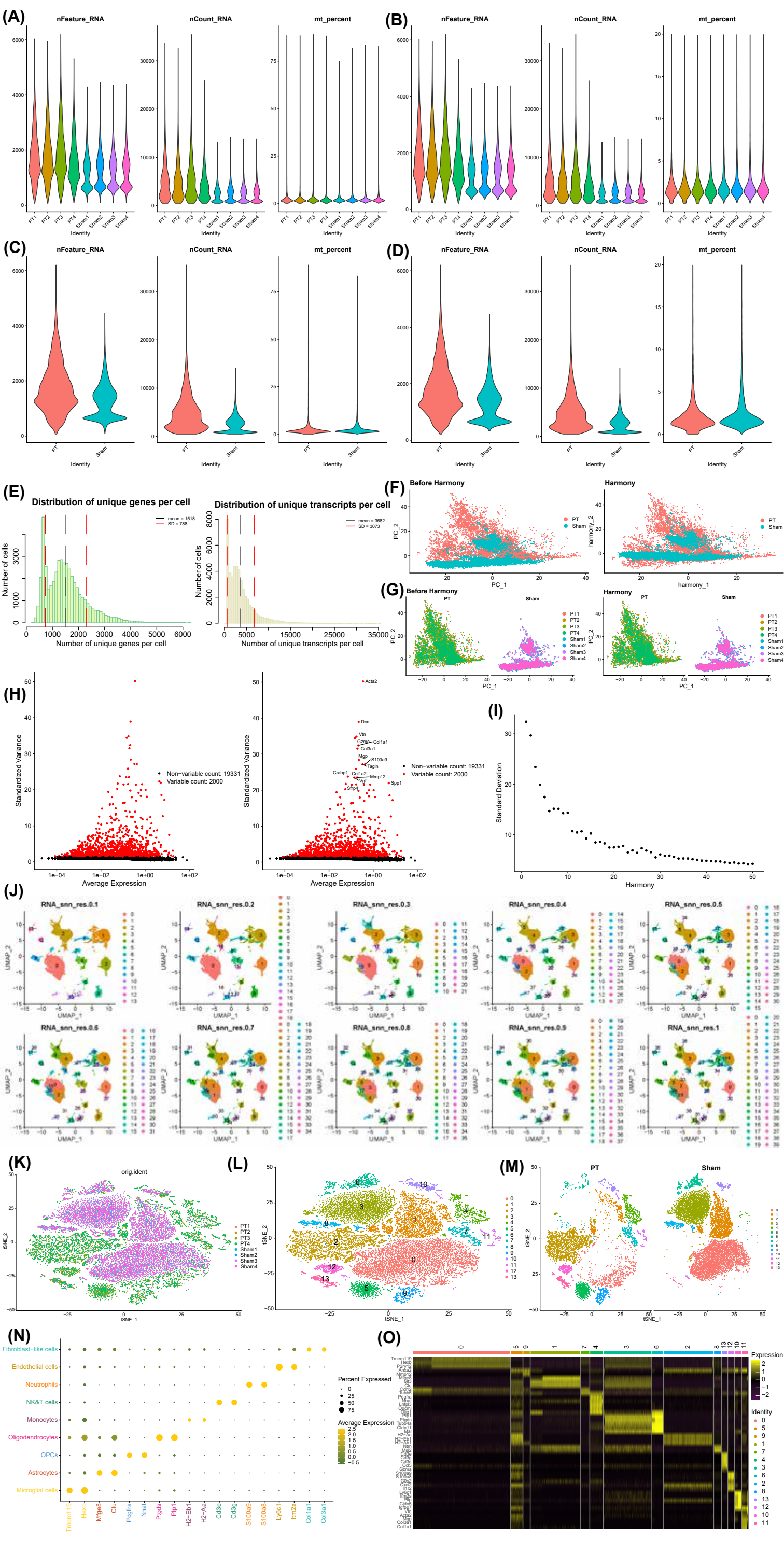

Supplement: Supporting Information 9 — Figure S1. Quality control, normalization, clustering of scRNA-seq data, and cell annotation. (A–D) Violin plots displaying the number of RNA features (nFeature_RNA), absolute UMI counts (nCount_RNA), and percentage of mitochondrial genes (mt_percent) before and after quality control filtering of each sequencing cell in the sham and PT groups. (E) Histogram showing the distribution of unique genes and transcripts per cell. (F–G) Dimensionality reduction plots displaying the results before and after Harmony integration of each sequencing cell in the sham and PT groups. (H) Volcano plots showing the high variable genes (HVGs). (I) Elbow plots of principal component analysis (PCA). (J) UMAP plots showing the different clusters at varying resolutions. (K–M) T-SNE plots showing the 13 clusters at a resolution of 0.1. (M) Bubble plots displaying the top two marker genes for each cell type. (N) Heatmap showing the top five marker genes for each cell type. [file 5828665.f9.pdf]

**(A)**

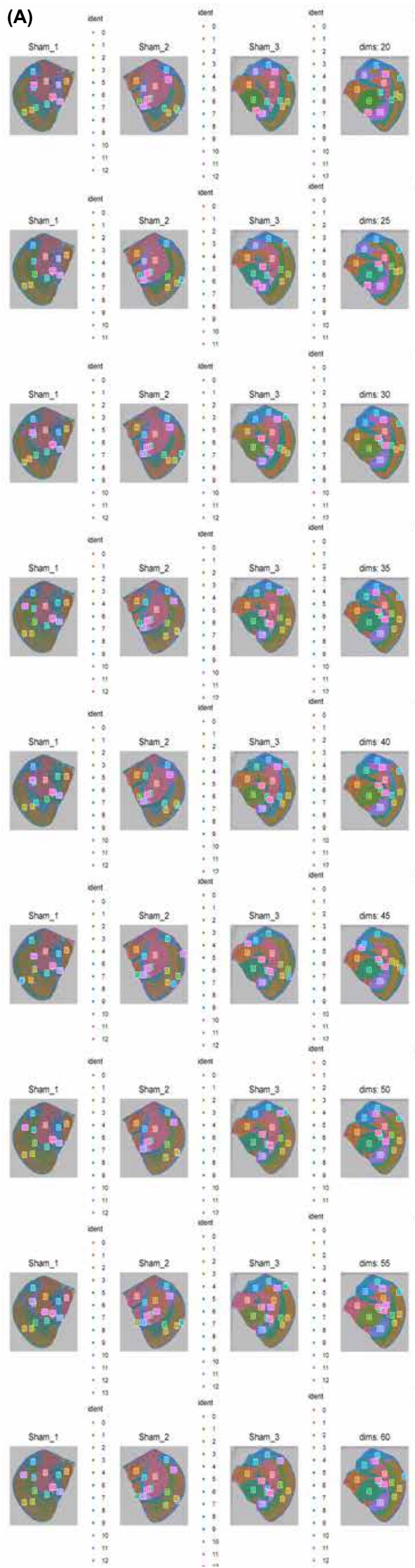

**(B)**

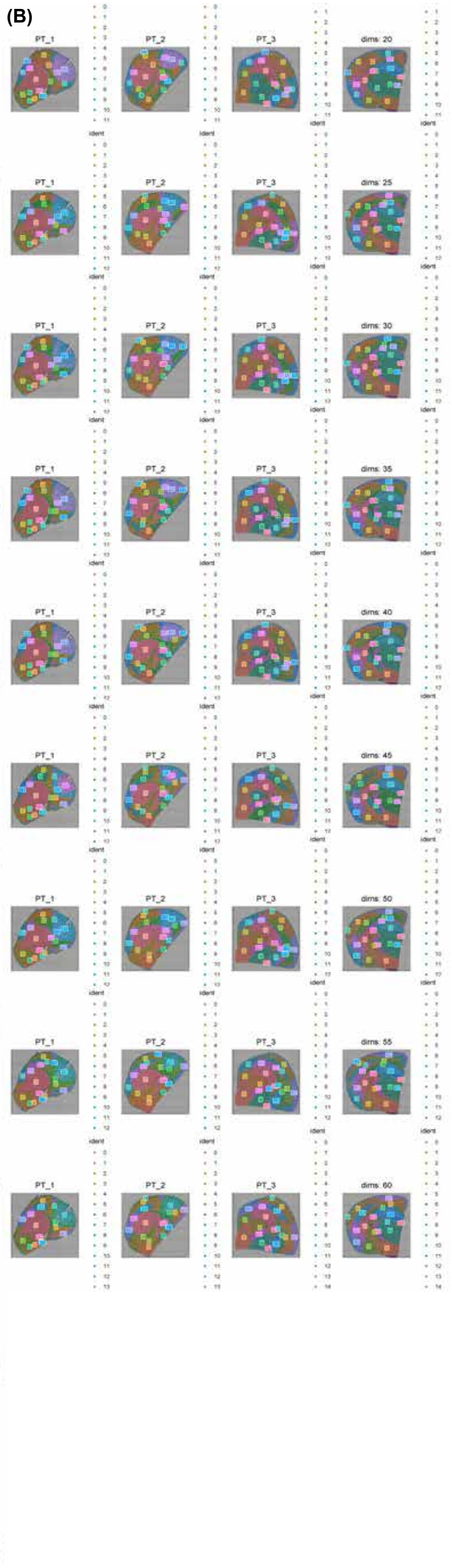

Supplement: Supporting Information 10 — Figure S2. Normalization of stRNA-seq data. (A, B) Normalization and integration of the stRNA-seq data using canonical correlation analysis (CCA) in the sham and PT groups. [file 5828665.f10.pdf]

(A)

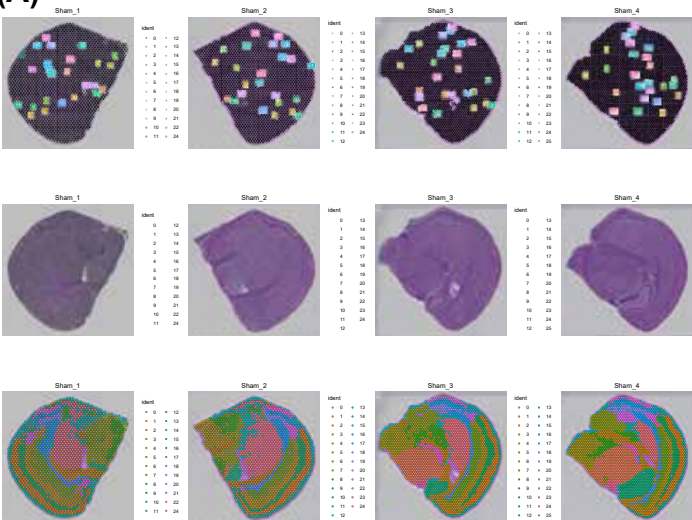

(B)

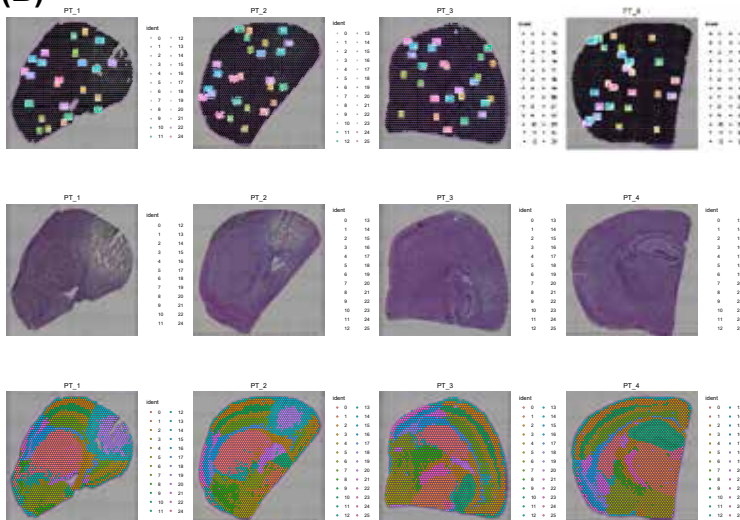

(C)

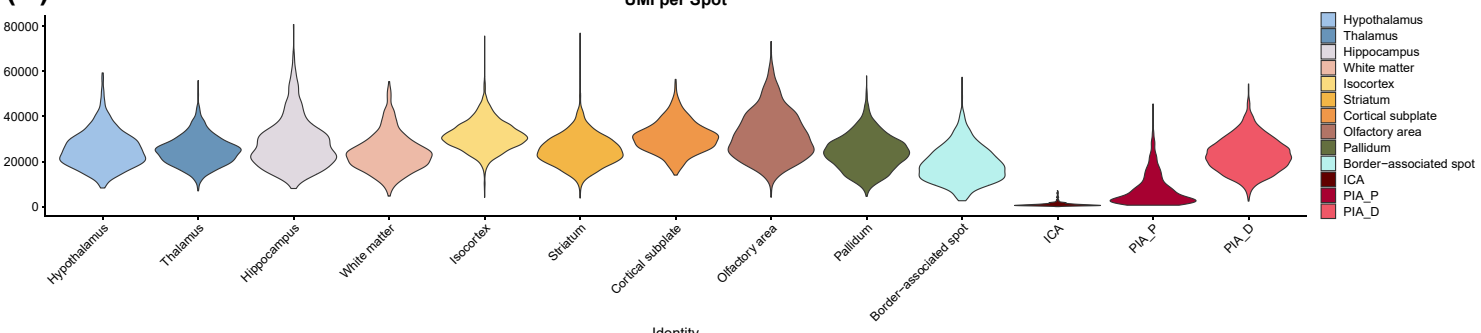

(D)

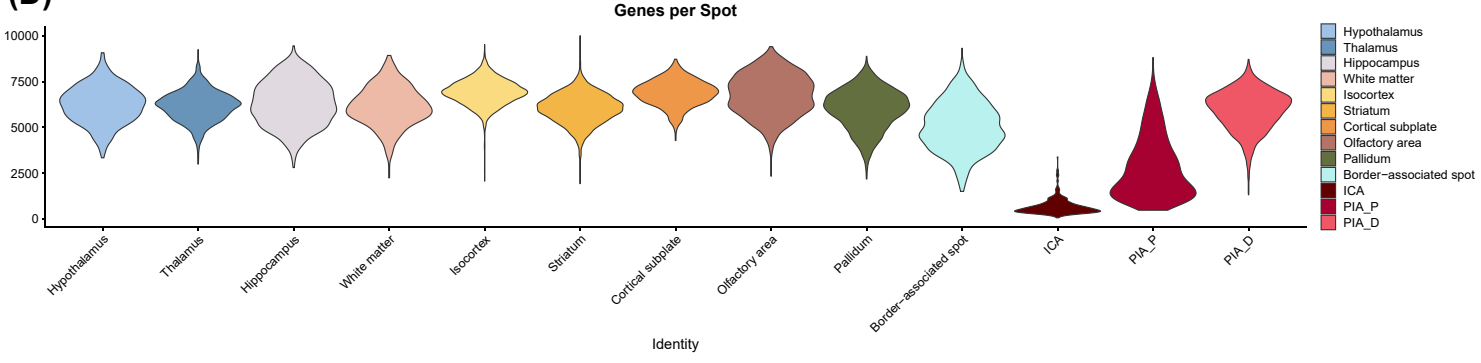

Supplement: Supporting Information 12 — Figure S4. Annotation of the niches in stRNA-seq data, and identification of fourteen brain regions and specific cell types in each region. (A,B) Clustering of 25 niches using 30 principal components (PCs) and a resolution of 0.8 in the sham and PT groups. (C,D) Violin plots displaying the UMI counts per spot and genes per spot in different regions. (E) H and E staining of mouse brain sections from the sham (n = 4) and PT (n = 4) groups, with transcriptome sections. (F) Spot clustering and annotation in the stRNA-seq data. (G) MIA heatmap showing the correlation significance analysis between stRNA-seq data and cell subsets from scRNA-seq data. −Log10 (phyper) is equivalent to −log10 (p-value). (H) Mapping of cell types from scRNA-seq data to stRNA-seq data using the “FindIntegrationAnchors” function. [file 5828665.f12.pdf]

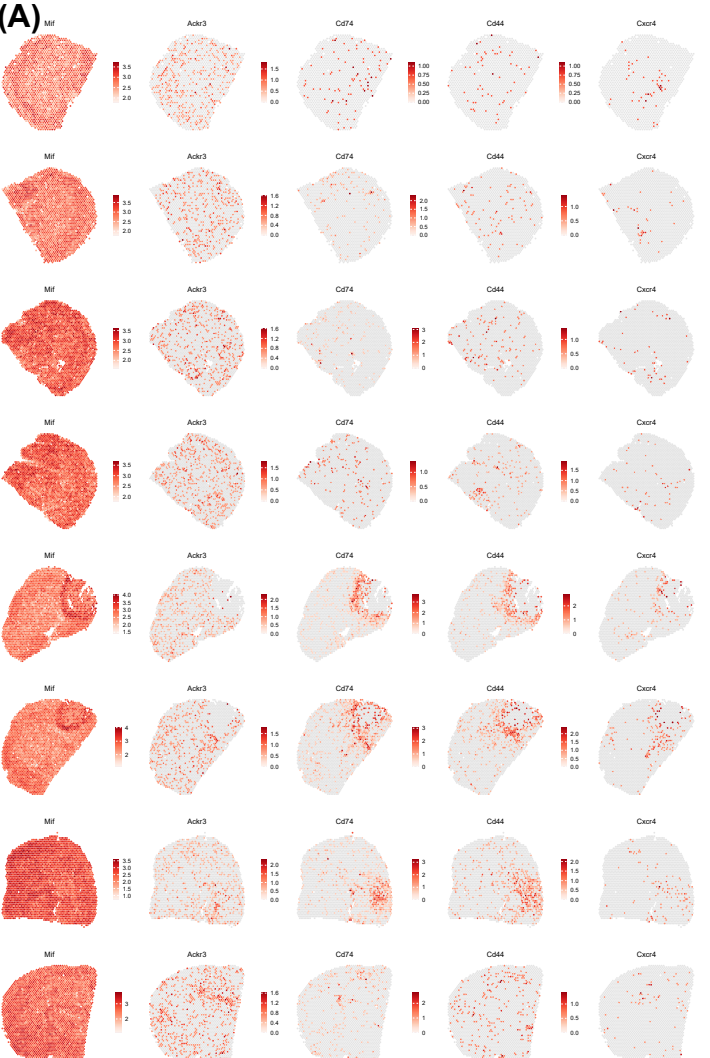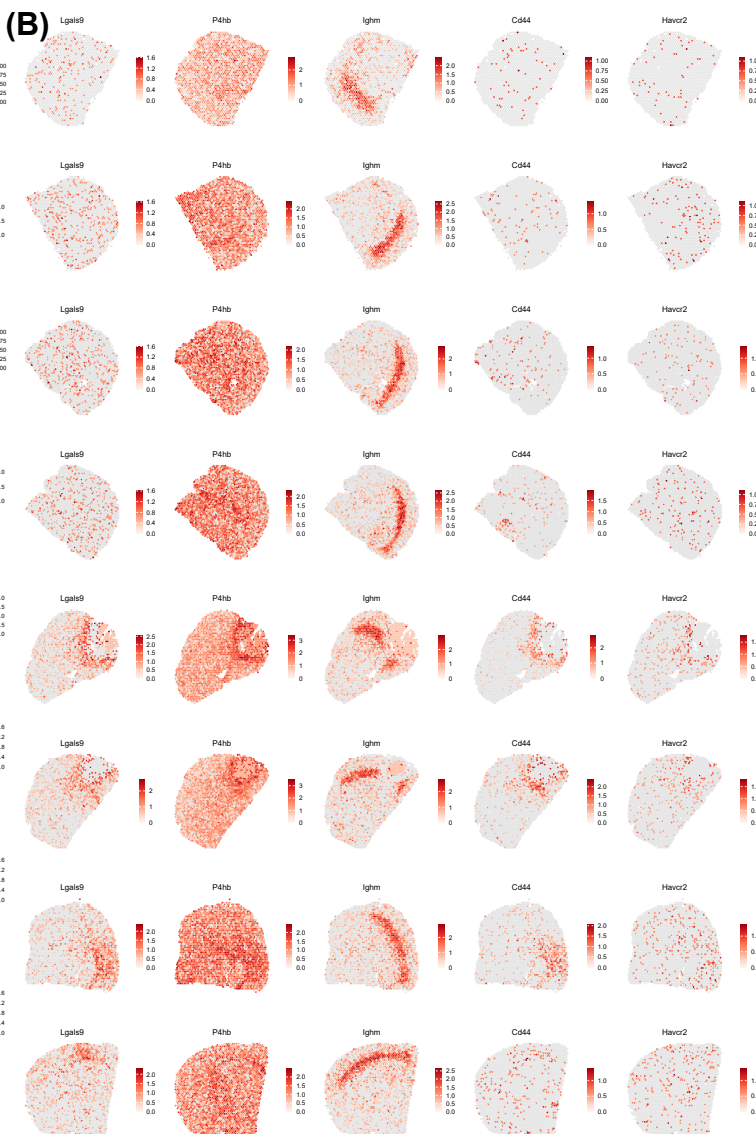

Supplement: Supporting Information 13 — Figure S5. Expression of ligand–receptor pairs across different spatial regions. (A) Expression of the MIF-ACKR3, MIF-CD74, MIF-CD44, and MIF-CXCR4 pairs in different spatial regions in the sham and PT groups. (B) Expression of the LGALS9-P4HB, LGALS9-LGHM, LGALS9-CD44, and LGALS9-HAVCR2 pairs in different spatial regions in the sham and PT groups. [file 5828665.f13.pdf]

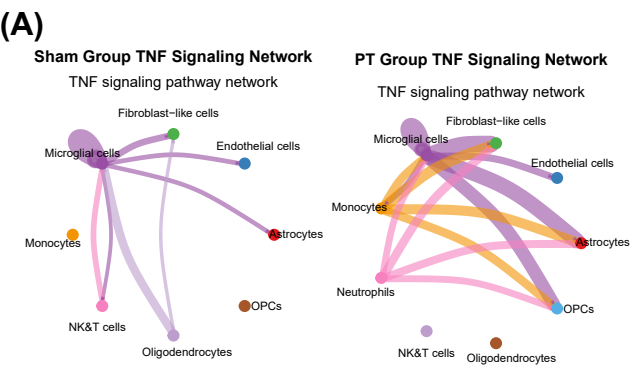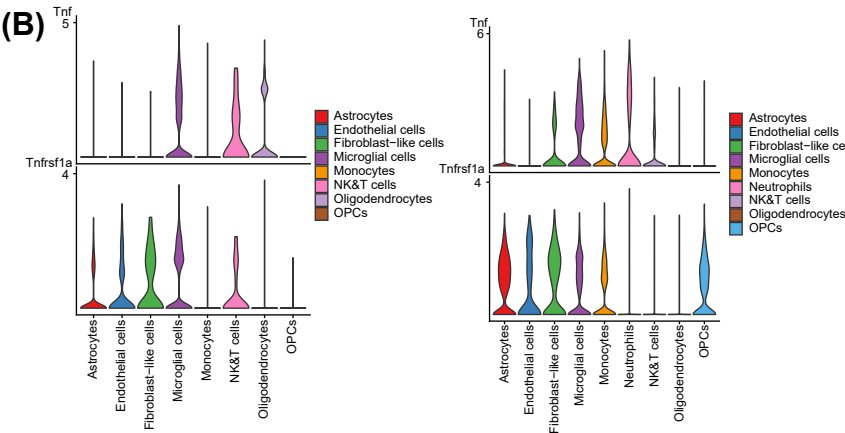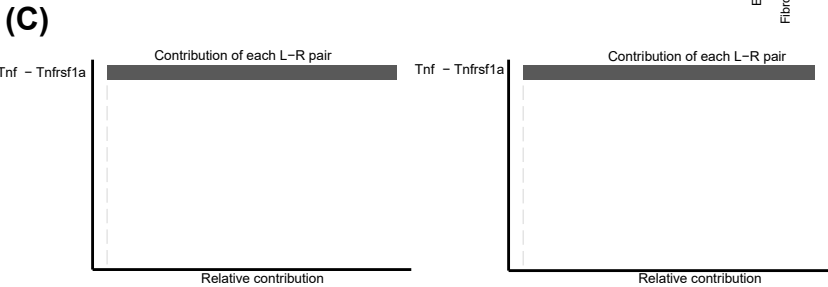

Supplement: Supporting Information 16 — Figure S8. TNF significant pathways in intercellular communications in the mouse brain following focal cortical ischemia. (A) Network displaying the predicted interaction strength of the TNF ligand–receptor pair in the sham and PT groups. (B) Violin plot showing the expression of the TNF ligand–receptor pair across different cell types in the sham and PT groups. (C) Histogram illustrating the contributions of the TNF ligand–receptor pair in various cell types in the sham and PT groups. [file 5828665.f16.pdf]
