# Supplementary material for: Comprehensive Analysis of the PANoptosis-Related Genes in Stroke Based on Single-Cell RNA-Seq and Spatial Transcriptomics
Source: Mediators Inflamm. 2025 Nov 4;2025:5828665. doi: 10.1155/mi/5828665 (PMC12605869; doi:10.1155/mi/5828665)
Supplement: Supporting Information 14 — Figure S6. Expression of module-specific hub genes across different spatial regions. (A, B) The average expression of module-specific hub genes across different spatial regions in the sham and PT groups, based on gene scores calculated by the UCell algorithm for the top 25 hub genes by kME in each module. [file 5828665.f14.pdf]

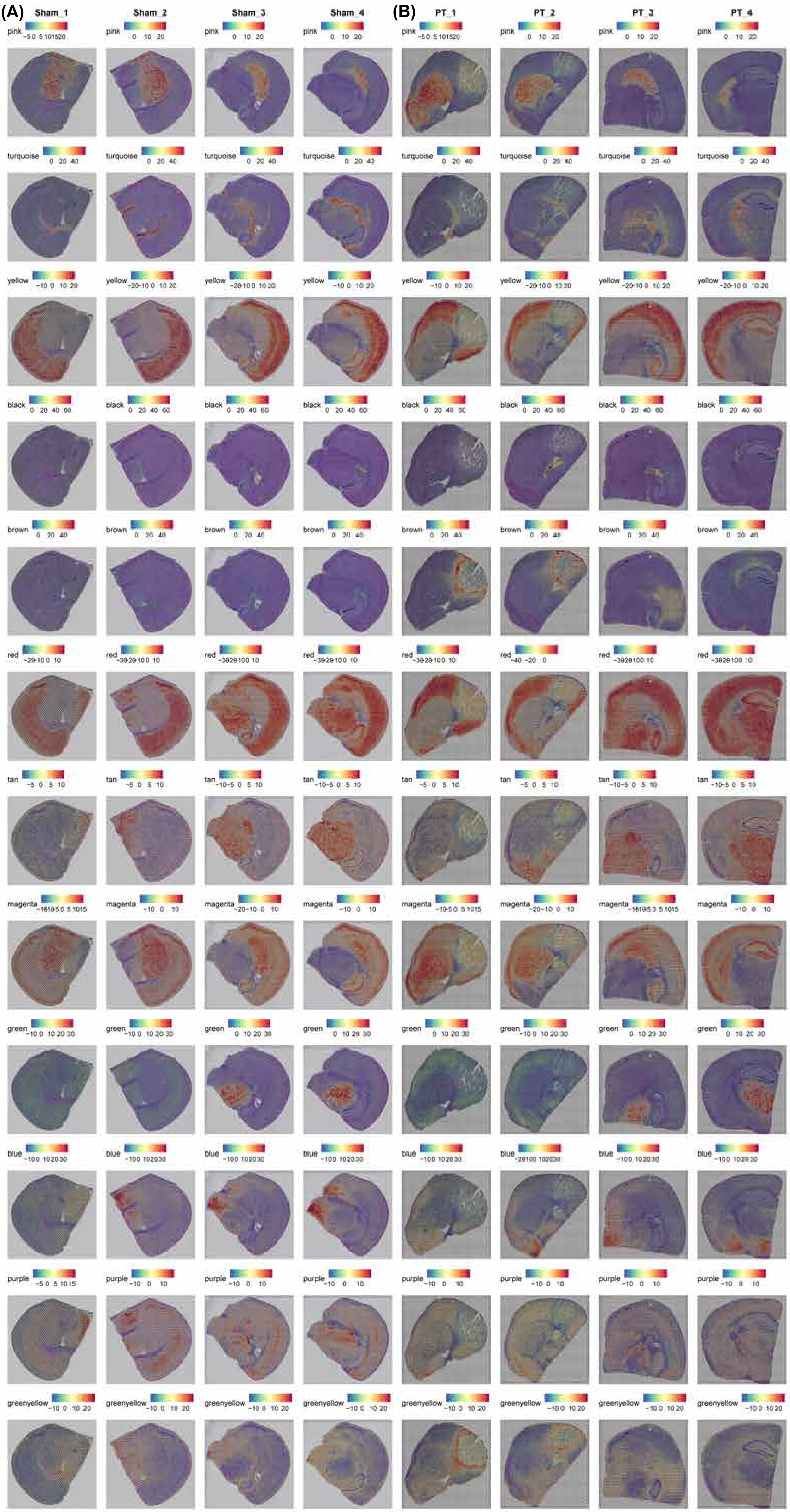
(A)
Sham_1
Sham_2
Sham_3
Sham_4
(B)
PT_1
PT_2
PT_3
PT_4
pink
turquoise
yellow
black
brown
red
tan
magenta
green
blue
purple
greenyellow
