# Supplementary material for: Comprehensive Analysis of the PANoptosis-Related Genes in Stroke Based on Single-Cell RNA-Seq and Spatial Transcriptomics
Source: Mediators Inflamm. 2025 Nov 4;2025:5828665. doi: 10.1155/mi/5828665 (PMC12605869; doi:10.1155/mi/5828665)
Supplement: Supporting Information 17 — Figure S9. Identification of the transcription factors (TFs) of MCL1, TNFRSF1A, and STAT3 using the “RcisTarget” package. (A) Global mean and standard deviation estimation. Shows the mean and standard deviation estimates for global gene recovery. (B) Enrichment analysis of the motif dbcorrdb__STAT3__ENCSR000DOQ_1__m1 in the gene set. (C) Enrichment analysis of the motif dbcorrdb__STAT3__ENCSR000DOX_1__m1 in the gene set. (D) Enrichment analysis of the motif taipale_tf_pairs__ETV2_DLX3_RSCGGAANNNNNNYAATTA_CAP in the gene set. (E) The top ten enriched motifs for MCL1, TNFRSF1A, and STAT3 and their corresponding transcription factors (TFs). [file 5828665.f17.pdf]

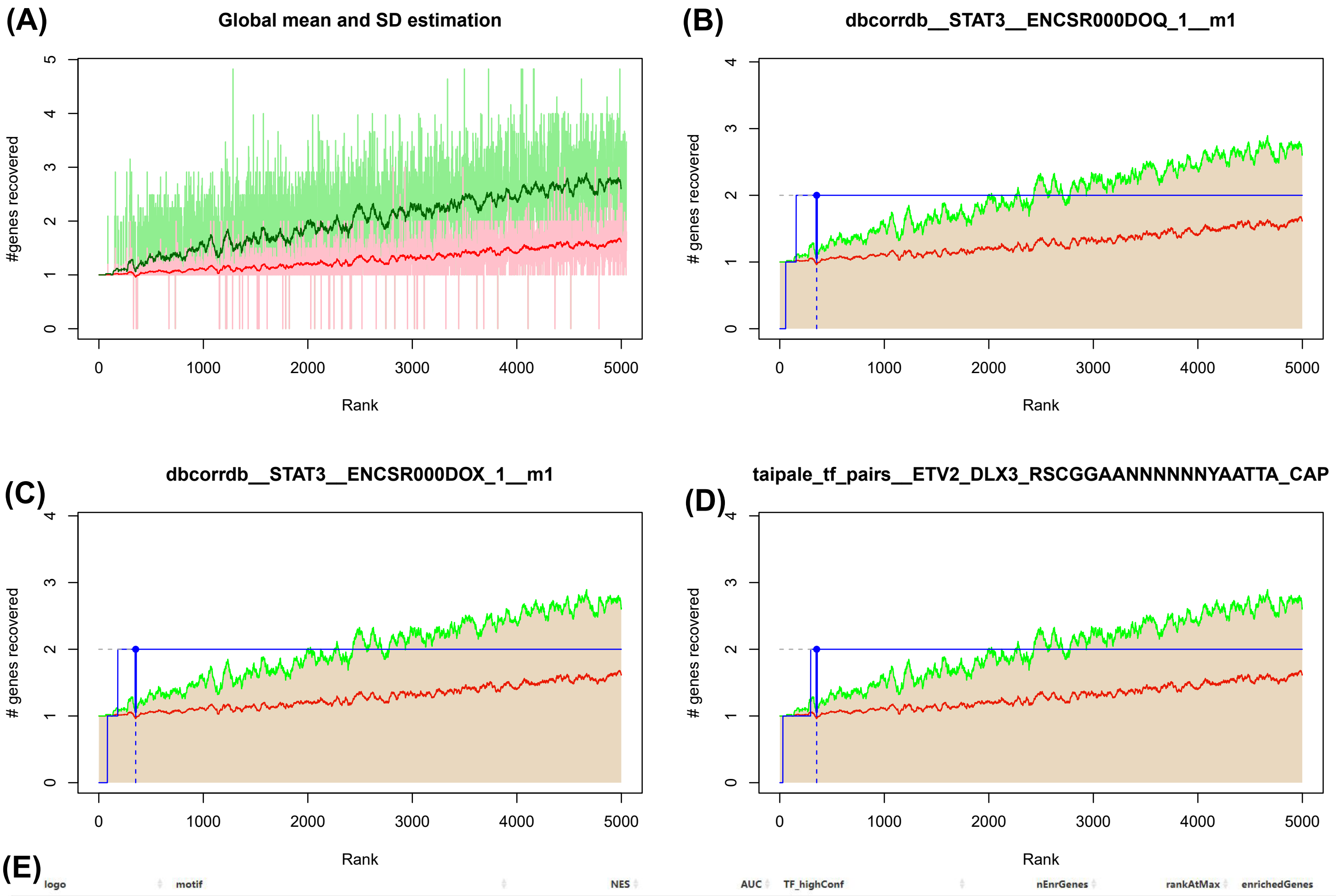

(E)

	logo	motif	NES	AUC	TF_highConf	nEnrGenes	rankAtMax	enrichedGenes
	All	All	All	All	All	All	All	All
1		predrem__nrMotif37	8.37	0.6		2	116	Stat3;Tnfrsf1a
2		dbcorrdb__STAT3__ENCSR000DOQ_1__m1	7.9	0.568	Stat3 (inferredBy_Orthology).	2	353	Stat3;Tnfrsf1a
3		swissregulon__sacCer__RGT1	7.61	0.548		2	353	Stat3;Tnfrsf1a
4		dbcorrdb__STAT3__ENCSR000DOX_1__m1	7.56	0.545	Stat3 (inferredBy_Orthology).	2	353	Stat3;Tnfrsf1a
5		predrem__nrMotif736	7.55	0.544		2	353	Stat3;Tnfrsf1a
6		transfac_pro__M01681	7.44	0.536		2	353	Stat3;Tnfrsf1a
7		taipale_tf_pairs__ETV2_DLX3_RSCGGAANNNNNNYAATTA_CAP	7.15	0.517	Dlx3; Etv2 (inferredBy_Orthology).	2	353	Stat3;Tnfrsf1a
8		cisbp__M4548	7.15	0.516	Stat3 (inferredBy_Orthology).	2	353	Stat3;Tnfrsf1a
9		dbcorrdb__STAT3__ENCSR000DOZ_1__m1	7.01	0.506	Stat3 (inferredBy_Orthology).	2	353	Stat3;Tnfrsf1a
10		predrem__nrMotif1897	6.92	0.5		2	353	Mcl1;Stat3
