# Supplementary material for: Comprehensive Analysis of the PANoptosis-Related Genes in Stroke Based on Single-Cell RNA-Seq and Spatial Transcriptomics
Source: Mediators Inflamm. 2025 Nov 4;2025:5828665. doi: 10.1155/mi/5828665 (PMC12605869; doi:10.1155/mi/5828665)
Supplement: Supporting Information 11 — Figure S3. Clustering of stRNA-seq data. (A, B) Dimensionality reduction by PCA. [file 5828665.f11.pdf]

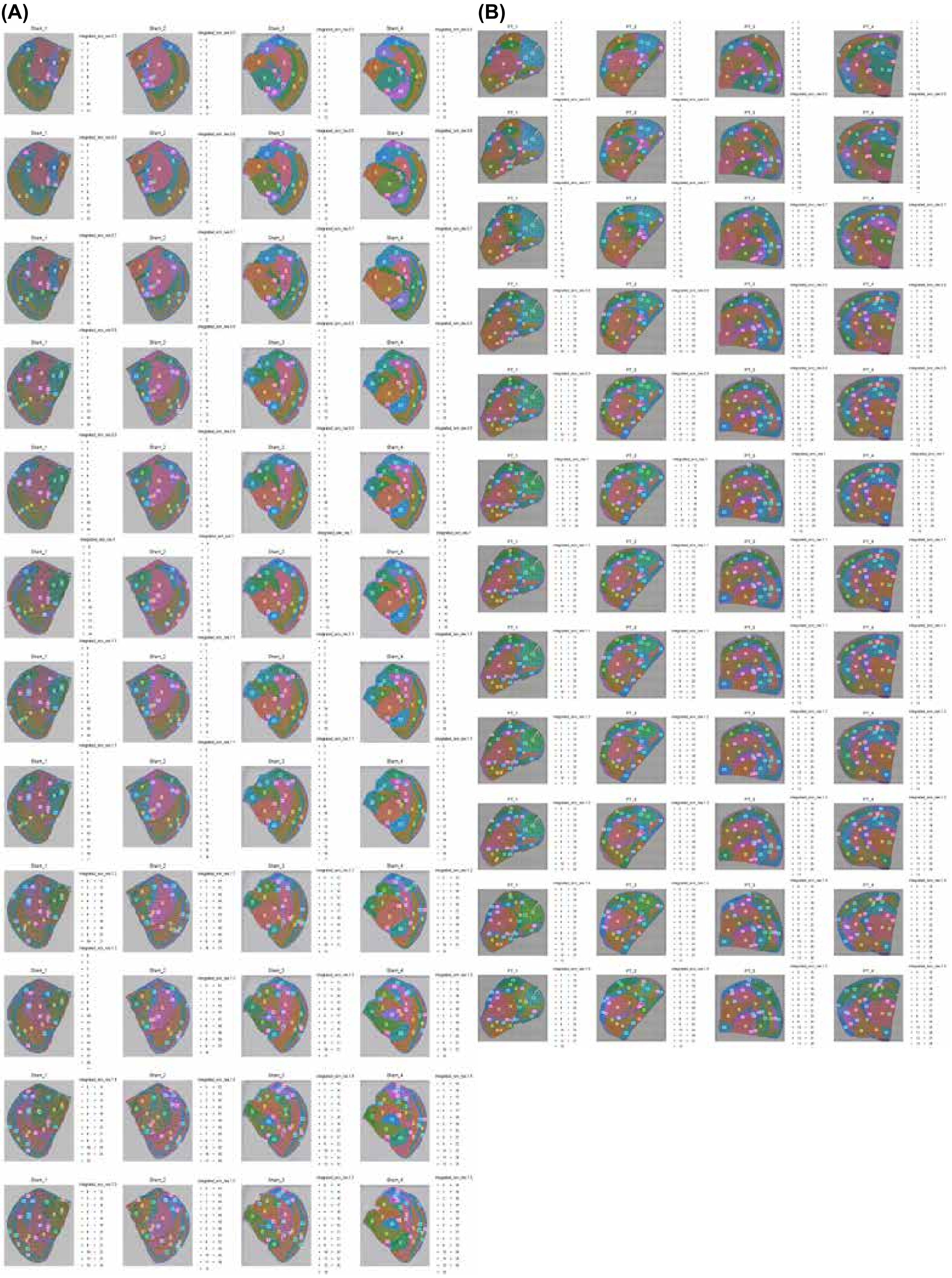
(A)
(B)
Sham_1
Sham_2
Sham_3
Sham_4
PT_1
PT_2
PT_3
PT_4
