# Supplementary material for: Comprehensive Analysis of the PANoptosis-Related Genes in Stroke Based on Single-Cell RNA-Seq and Spatial Transcriptomics
Source: Mediators Inflamm. 2025 Nov 4;2025:5828665. doi: 10.1155/mi/5828665 (PMC12605869; doi:10.1155/mi/5828665)
Supplement: Supporting Information 15 — Figure S7. Gene Set Enrichment Analysis (GSEA). (A) The top 30 enriched pathways for MCL1 in the stroke and sham groups. (B) The top 30 enriched pathways for STAT3 in the stroke and sham groups. (C) The top 30 enriched pathways for TNFRSF1A in the stroke and sham groups. Red represents the p-value, and the size of the dot represents the number of enriched genes. [file 5828665.f15.pdf]

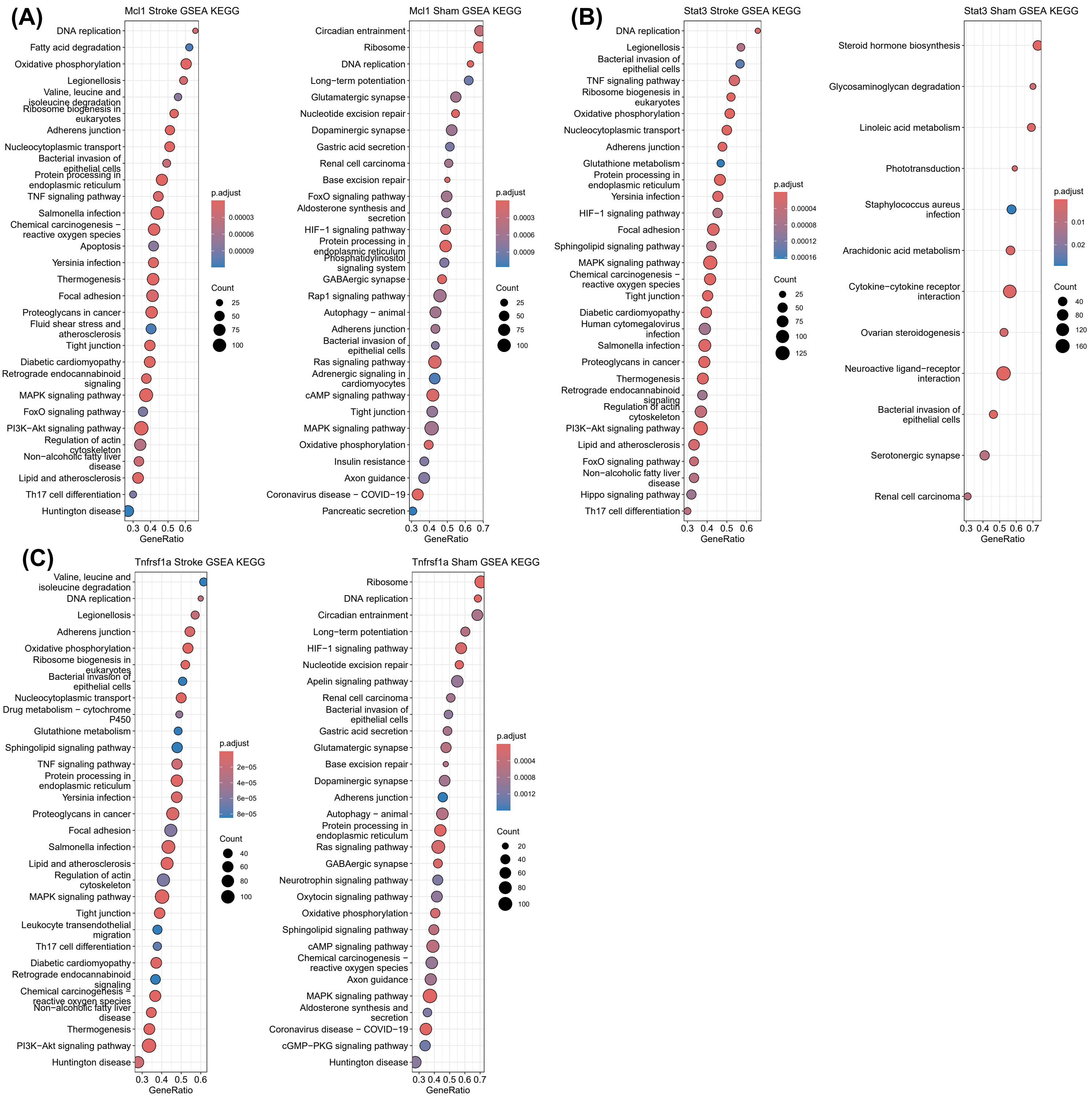
(A)
Mcl1 Stroke GSEA KEGG
DNA replication
Fatty acid degradation
Oxidative phosphorylation
Legionellosis
Valine, leucine and isoleucine degradation
Ribosome biogenesis in eukaryotes
Adherens junction
Nucleocytoplasmic transport
Bacterial invasion of epithelial cells
Protein processing in endoplasmic reticulum
TNF signaling pathway
Salmonella infection
Chemical carcinogenesis − reactive oxygen species
Apoptosis
Yersinia infection
Thermogenesis
Focal adhesion
Proteoglycans in cancer
Fluid shear stress and atherosclerosis
Tight junction
Diabetic cardiomyopathy
Retrograde endocannabinoid signaling
MAPK signaling pathway
FoxO signaling pathway
PI3K−Akt signaling pathway
Regulation of actin cytoskeleton
Non−alcoholic fatty liver disease
Lipid and atherosclerosis
Th17 cell differentiation
Huntington disease
0.3 0.4 0.5 0.6
GeneRatio
p.adjust
0.00003
0.00006
0.00009
Count
25
50
75
100
Mcl1 Sham GSEA KEGG
Circadian entrainment
Ribosome
DNA replication
Long−term potentiation
Glutamatergic synapse
Nucleotide excision repair
Dopaminergic synapse
Gastric acid secretion
Renal cell carcinoma
Base excision repair
FoxO signaling pathway
Aldosterone synthesis and secretion
HIF−1 signaling pathway
Protein processing in endoplasmic reticulum
Phosphatidylinositol signaling system
GABAergic synapse
Rap1 signaling pathway
Autophagy − animal
Adherens junction
Bacterial invasion of epithelial cells
Ras signaling pathway
Adrenergic signaling in cardiomyocytes
cAMP signaling pathway
Tight junction
MAPK signaling pathway
Oxidative phosphorylation
Insulin resistance
Axon guidance
Coronavirus disease − COVID−19
Pancreatic cancer secretion
0.3 0.4 0.5 0.6 0.7
GeneRatio
p.adjust
0.0003
0.0006
0.0009
Count
25
50
75
100
(B)
Stat3 Stroke GSEA KEGG
DNA replication
Legionellosis
Bacterial invasion of epithelial cells
TNF signaling pathway
Ribosome biogenesis in eukaryotes
Oxidative phosphorylation
Nucleocytoplasmic transport
Adherens junction
Glutathione metabolism
Protein processing in endoplasmic reticulum
Yersinia infection
HIF−1 signaling pathway
Focal adhesion
Sphingolipid signaling pathway
MAPK signaling pathway
Chemical carcinogenesis − reactive oxygen species
Tight junction
Diabetic cardiomyopathy
Human cytomegalovirus infection
Salmonella infection
Proteoglycans in cancer
Thermogenesis
Retrograde endocannabinoid signaling
Regulation of actin cytoskeleton
PI3K−Akt signaling pathway
Lipid and atherosclerosis
FoxO signaling pathway
Non−alcoholic fatty liver disease
Hippo signaling pathway
Th17 cell differentiation
0.3 0.4 0.5 0.6
GeneRatio
p.adjust
0.00004
0.00008
0.00012
0.00016
Count
25
50
75
100
125
Stat3 Sham GSEA KEGG
Steroid hormone biosynthesis
Glycosaminoglycan degradation
Linoleic acid metabolism
Phototransduction
Staphylococcus aureus infection
Arachidonic acid metabolism
Cytokine−cytokine receptor interaction
Ovarian steroidogenesis
Neuroactive ligand−receptor interaction
Bacterial invasion of epithelial cells
Serotonergic synapse
Renal cell carcinoma
0.3 0.4 0.5 0.6 0.7
GeneRatio
p.adjust
0.01
0.02
Count
40
80
120
160
(C)
Tnfrsf1a Stroke GSEA KEGG
Valine, leucine and isoleucine degradation
DNA replication
Legionellosis
Adherens junction
Oxidative phosphorylation
Ribosome biogenesis in eukaryotes
Bacterial invasion of epithelial cells
Nucleocytoplasmic transport
Drug metabolism − cytochrome P450
Glutathione metabolism
Sphingolipid signaling pathway
TNF signaling pathway
Protein processing in endoplasmic reticulum
Yersinia infection
Proteoglycans in cancer
Focal adhesion
Salmonella infection
Lipid and atherosclerosis
Regulation of actin cytoskeleton
MAPK signaling pathway
Tight junction
Leukocyte transendothelial migration
Th17 cell differentiation
Diabetic cardiomyopathy
Retrograde endocannabinoid signaling
Chemical carcinogenesis − reactive oxygen species
Non−alcoholic fatty liver disease
Thermogenesis
PI3K−Akt signaling pathway
Huntington disease
0.3 0.4 0.5 0.6
GeneRatio
p.adjust
2e−05
4e−05
6e−05
8e−05
Count
40
60
80
100
Tnfrsf1a Sham GSEA KEGG
Ribosome
DNA replication
Circadian entrainment
Long−term potentiation
HIF−1 signaling pathway
Nucleotide excision repair
Apelin signaling pathway
Renal cell carcinoma
Bacterial invasion of epithelial cells
Gastric acid secretion
Glutamatergic synapse
Base excision repair
Dopaminergic synapse
Adherens junction
Autophagy − animal
Protein processing in endoplasmic reticulum
Ras signaling pathway
GABAergic synapse
Neurotrophin signaling pathway
Oxytocin signaling pathway
Oxidative phosphorylation
Sphingolipid signaling pathway
cAMP signaling pathway
Chemical carcinogenesis − reactive oxygen species
Axon guidance
MAPK signaling pathway
Aldosterone synthesis and secretion
Coronavirus disease − COVID−19
cGMP−PKG signaling pathway
Huntington disease
0.3 0.4 0.5 0.6 0.7
GeneRatio
p.adjust
0.0004
0.0008
0.0012
Count
20
40
60
80
100
